# Navigating Pompe Disease Assessment: A Comprehensive Scoping Review

**DOI:** 10.7759/cureus.73593

**Published:** 2024-11-13

**Authors:** Leticia Nunes Campos, Israel Davila Rivera, Daiana M Ibañez Alegre, Fabiana N del Puerto González, Mónica Garrido San Juan, Federico Fernandez Zelcer, Delfina Borgobello, Ayla Gerk, Laura F Sosa, Marcos M Miretti, Jorgelina Stegmann, Carina F Argüelles

**Affiliations:** 1 Rare Diseases, Rare Diseases Community (RDCom), Buenos Aires, ARG; 2 Genetics, GIGA, Instituto de Biología Subtropical, Nodo Posadas, Universidad Nacional de Misiones (UNaM) - Consejo Nacional de Investigaciones Científicas y Técnicas (CONICET), Posadas, ARG; 3 Faculty of Philosophy and Letters, Universidad de Buenos Aires, Buenos Aires, ARG; 4 Faculty of Health Sciences, Universidad Católica de las Misiones, Posadas, ARG

**Keywords:** cardiomyopathy, hypertrophic, lysosomal storage diseases, muscle weakness, rare diseases, storage disease type ii

## Abstract

Pompe disease (PD) is a rare progressive autosomal recessive disorder resulting from the deficiency of acid alpha-glucosidase (GAA) enzyme activity. Due to its multisystemic involvement, PD leads to significant morbidity and impacts patients' quality of life. Despite the availability of approved disease-modifying treatments, the prompt diagnosis and management of PD, which are crucial for patient outcomes, still present several challenges. This scoping review aimed to synthesize the evidence regarding methods for screening, diagnosing, and following up PD. We searched articles in English and Spanish published from 2017 to February 8, 2022, across 11 databases (i.e., Cochrane Database of Systematic Reviews, Directory of Open Access Journals (DOAJ), Epistemonikos, Ingenta Connect, Medline/PubMed, SAGE, SciELO Citation Index, ScienceDirect, Springer Link, Virtual Health Library, and Wiley Online Library). We included primary studies (i.e., case reports, case series, cross-sectional studies, case controls, cohorts, clinical trials, and qualitative studies), reviews, and guidelines that described at least one assessment method for patients with confirmed clinical, genetic, or biochemical PD. Two independent reviewers screened and extracted data from articles, with a third reviewer solving conflicts. We synthesized data with narrative summaries and descriptive statistics. After screening 2,139 titles and abstracts, we included 96 eligible articles. Cross-sectional studies (n = 30) and guidelines (n = 1) were the most and least prevalent designs, respectively. Most studies targeted late-onset PD (LOPD, n = 48) and infantile-onset PD (IOPD, n = 21). Eleven articles described newborn screening programs, highlighting their potential to improve PD prevalence estimations and still limited availability among countries. Overall, 81 articles documented clinical manifestations of PD. Hypotonia (n = 7) and hypertrophic cardiomyopathy (n = 7) were the most documented for IOPD, while progressive muscle weakness (n = 21) and dyspnea (n = 11) were the most prevalent for LOPD. We found 26 articles reporting biochemical assays, with dried blood spots (DBS) for GAA enzyme deficiency detection being the most cited (n = 19). We also noted a lack of standardization in documenting DBS results. Additionally, 21 articles mentioned genetic studies, with next-generation sequencing emerging as the gold standard for identifying mutated alleles. Functional studies were the most utilized to follow up with patients. However, monitoring strategies for pediatric and adult PD lacked consensus, and only one article assessed patients' quality of life. This review comprehensively evaluated the literature on PD screening, diagnosis, and follow-up methods, identifying prevalent techniques within each assessment category. We emphasized the need for a more standardized approach to reporting biochemical assays, genetic testing, and clinical presentations. Our review also underscored the critical lack of standardization in PD follow-up. Addressing these gaps will enhance the comparability of future research findings and improve the quality of PD-related healthcare. Limitations of this review included restricting eligible languages and publication years to the latest five, the methodological heterogeneity of selected articles, and the lack of individual study bias assessment.

## Introduction and background

Pompe disease (PD), also known as glycogen storage disease type II (GSDII; ORPHA:365; ORPHA:308552; MONDO:0009290; MIM #232300 and MIM#606800) is a rare monogenic, autosomal recessive metabolic disorder caused by mutations in the acid alpha-glucosidase (GAA) gene located at 17q25.2-q25.3 [1,2]. These mutations cause a deficient production of the GAA protein, leading to lysosomal glycogen accumulation in the nervous system in several tissues, particularly in skeletal and cardiac muscles. Consequently, this accumulation causes cellular dysfunction and muscle damage [1,2]. To date, more than 600 mutations associated with the GAA gene have been identified [3,4], some of which are prevalent in specific ethnic groups and geographic regions [5].

PD is clinically heterogeneous and generally classified into two main phenotypes based on age of onset, progression rate, severity, and organ involvement: infantile-onset PD (IOPD) and late-onset PD (LOPD) [6,7]. IOPD (ORPHA:308552) is the most severe phenotype, characterized by rapidly progressive hypertrophic cardiomyopathy and generalized muscle weakness at birth, often resulting in mortality within the first two years of life [6]. In contrast, LOPD (ORPHA:420429) encompasses childhood, juvenile, and adult-onset disease. Patients with LOPD typically exhibit variable muscle involvement, progressive proximal muscle weakness, and respiratory insufficiency, with onset ranging from infancy to the sixth decade of life [6,7]. LOPD progresses more slowly than IOPD and eventually affects the diaphragm and accessory respiratory muscles, resulting in respiratory failure, the most prevalent cause of mortality in this phenotype [8]. PD shares symptom overlap with several neuromuscular disorders, including facioscapulohumeral, Duchenne, and Becker muscular dystrophies.

Given the availability of approved disease-modifying treatments and their impact on patients’ quality of life (QOL), prompt PD screening, diagnosis, and follow-up are critical [9]. However, identifying PD presents several challenges, including variable phenotypes, limited awareness, and overlap with other neuromuscular disorders, such as limb-girdle muscular dystrophies [9,10]. Therefore, standardizing screening, diagnosis, and follow-up strategies in PD is imperative to improve patient outcomes.

With this review, we aimed to map and synthesize the existing evidence on assessment methods for screening, diagnosing, and monitoring patients with PD. By addressing the current challenges in PD assessment, this review seeks to highlight the importance of early and accurate diagnosis in optimizing patient care and outcomes.

We previously posted this article on the Research Square preprint platform on January 30, 2024.

## Review

Study design

In this scoping review, our primary aim was to identify the existing methods for assessing patients with PD worldwide. We defined "assessment" as understanding a patient's condition [11]. We chose to conduct a scoping review as it allows the evaluation of a broad range of research and the summarization of heterogeneous findings. Our protocol was registered with the Open Science Framework, and the study was reported following the Preferred Reporting Items for Systematic Reviews and Meta-Analysis extension for Scoping Reviews (PRISMA-ScR) [12,13].

This review considered methods for screening, diagnosing, and monitoring PD patients. Screening methods comprised tests, exams, and procedures to identify diseases in a targeted, usually asymptomatic population [14]. Diagnostic methods encompassed those used to determine the nature of a condition [15]. Follow-up or monitoring methods referred to those that assess long-term outcomes of PD [16]. Assessment methods included but were not limited to clinical manifestations, scales, questionnaires, and indices, along with genetic, imaging, biochemical, and electrophysiological studies.

Eligibility criteria

We considered articles that included patients who fulfilled clinical, genetic, or biochemical PD diagnostic criteria as previously mentioned [17,18]. Table 1 describes the features that raise clinical suspicion of PD. We included human-based research articles using qualitative or quantitative methods for assessing PD patients from any country. Eligible articles were primary studies (i.e., case reports, case series, cross-sectional studies, case-control studies, cohorts, clinical trials, qualitative studies), reviews (i.e., narrative, scoping, and systematic reviews), and guidelines.

**Table 1:** Clinical features of suspect Pompe disease phenotypes

Phenotype	
Infantile-onset Pompe disease (IOPD)	Late-onset Pompe disease (LOPD)
1. Cardiovascular system abnormalities:	1. Neuromuscular system abnormalities:
Cardiomegaly or hypertrophic cardiomyopathy	Progressive muscle weakness (limb-girdle, upper and lower limbs)
	Muscle wasting
2. Musculoskeletal system abnormalities:	Waist dystrophy
Rapidly progressive muscle weakness	Gait disorders
Hypotonia	Chewing and swallowing difficulties
Delayed or not achieved rolling over, sitting up, and standing	Drooping of the upper eyelids (ptosis)
3. Craniofacial manifestations abnormalities:	2. Respiratory system abnormalities:
Large, protruding tongue	Dysfunction of the diaphragm and intercostal muscles
	Restrictive respiratory insufficiency/varying degrees of respiratory weakness
4. Digestive system abnormalities:	3. Cardiovascular system abnormalities
Moderate enlargement of the liver	4. Digestive system abnormalities
Feeding and swallowing problems	5. Urinary system abnormalities
	6. Metabolic and endocrine system findings:
5. Respiratory system abnormalities:	Idiopathic hyperkalemia
Respiratory dysfunction (combined or not with respiratory tract infections)	7. Connective tissue and joint abnormalities:
6. Nervous system abnormalities:	Idiopathic scoliosis
Hearing loss	8. Rheumatological manifestations
	Non-specific chronic low back pain

As our exclusion criteria, we disregarded editorials, commentaries, and personal anecdotes because they primarily express opinions rather than present original findings. We also excluded conference abstracts and books due to their limited information on methods and since they typically undergo less rigorous scientific assessment than peer-reviewed articles. Additionally, we excluded studies focusing on pharmacological and non-pharmacological interventions, as the effects of specific therapeutics were not part of our scope. Animal and in vitro studies were also not considered since their evidence is not directly applicable to humans and clinical practice.

Information sources, search strategy, and citation management

A librarian (FFZ) conducted the search strategy at 11 databases: Cochrane Database of Systematic Reviews (John Wiley & Sons, Inc), Directory of Open Access Journals (DOAJ), Epistemonikos (Epistemonikos Foundation), Ingenta Connect (Ingenta), Medline/PubMed (National Library of Medicine, NCBI), SAGE (Sage Publications), SciELO Citation Index (Web of Science, Clarivate), ScienceDirect (Elsevier BV), Springer Link (Springer Nature Switzerland AG), Virtual Health Library (Latin American and Caribbean Center on Health Sciences Information, World Health Organization), and Wiley Online Library (John Wiley & Sons, Inc). We chose these databases due to their extensive coverage of biomedical literature across different contexts and populations, increasing the likelihood of retrieving relevant articles for our review. The search strategy included articles published between 2017 and the search date on February 8, 2022. This timeframe ensured we encompassed contemporary evidence of current practices and emerging trends in the field. We included articles published in English and Spanish due to the research team's language proficiency skills. Supplementary Material 1 provides the keywords and search strategy per database.

References were imported into the EndNote bibliography manager, and duplicated references were automatically purged by searching for simultaneous matches in the author, title, and year fields [19]. FFZ conducted an additional manual search to identify further duplicates. We exported the result from the search strategy as a Google Sheets (Google, Inc., Mountain View, CA) spreadsheet [20].

Study selection

We screened eligible studies using a standardized Google Form (Google, Inc., Mountain View, CA) to register reviewers' decisions [21]. Before the screening, we conducted a calibration exercise with reviewers (IDR, DMIA, FNDPG, MGSJ). Reviewers independently screened a random sample of 20 articles from the search. Screening commenced when reviewers achieved an agreement percentage equal to or greater than 90%. After calibration, the reviewers independently screened the articles' titles and abstracts following the eligibility criteria. Subsequently, reviewers screened the full text of the remaining studies to confirm eligibility. At each screening stage, CFA and LNC solved discrepancies between reviewers.

Data extraction

We created a Google Sheets spreadsheet to extract data from the included articles. Before starting data extraction, all reviewers participated in a calibration exercise to ensure consistency in data collection. Reviewers had to achieve a minimum agreement percentage of 90%. Two independent reviewers extracted data from each article, and CFA resolved disagreements. We extracted the following data points: title, abstract, authors' names, digital object identifier (DOI), year of publication, language used for publication, study design, number of study participants, patients' age, sex distribution, and country of study setting. We also collected the names of reported assessment methods per article, their category (e.g., clinical, imaging, genetic), and findings. We did not conduct any risk of bias assessment, consistent with the scoping review methodology [22,23].

Synthesis of results

We used frequencies and percentages to summarize the characteristics of the included articles (e.g., study design, country where studies were conducted, number of participants) and quantify how often articles reported each assessment method and clinical manifestation. For each major category (e.g., biochemical assays, genetic tests, imaging studies), we structured a narrative summary of the findings from reported methods by condensing the key themes and patterns observed across the studies. We also presented our results with tables and figures prepared using Microsoft Excel 365 (Microsoft Corporation, Redmond, WA).

Literature search

**Figure 1:**
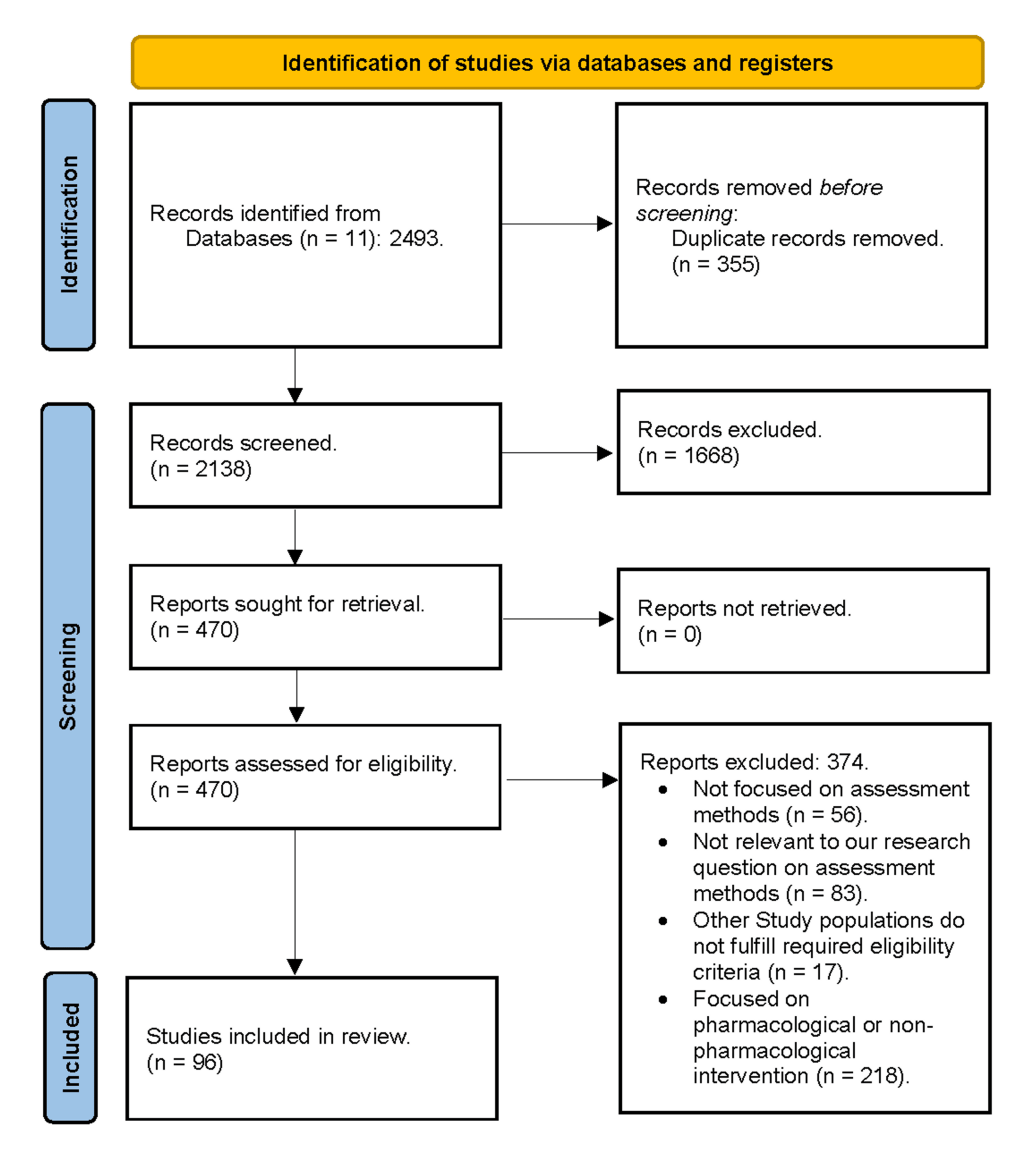
Preferred Reporting Items for Systematic Reviews and Meta-Analysis (PRISMA) flowchart summarizing the study selection process The flowchart outlines the flow of information by mapping the number of items identified, included, and excluded per screening stage.

The literature search yielded 2,494 articles. After removing 355 duplicates, 2,139 articles underwent title and abstract screening. Among these, 470 articles underwent full-text screening. Ninety-six articles met the eligibility criteria (Figure 1 and Supplementary Material 2). 

Characteristics of selected articles

**Figure 2:**
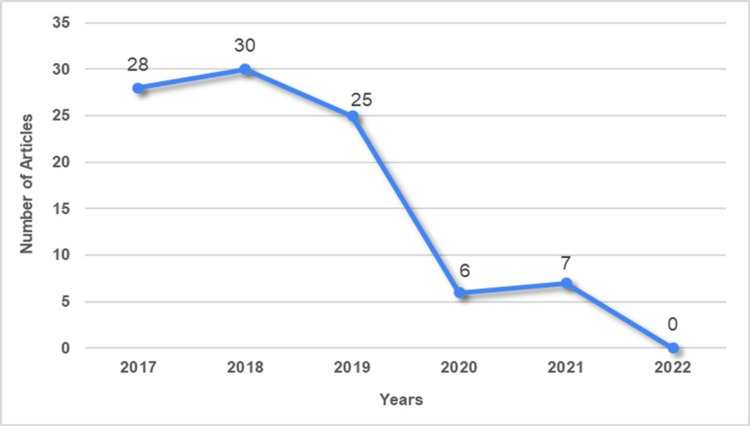
Number of included articles per year The figure shows the number of selected articles per year considering the eligible period of our scoping review (January 1, 2017-February 8, 2022).

**Figure 3:**
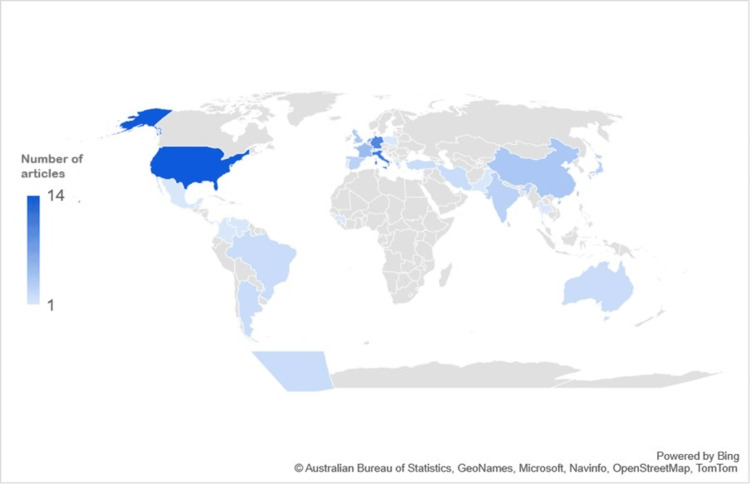
Number of included articles per country This world map depicts the number of included articles per country among the 96 selected articles.

**Figure 4:**
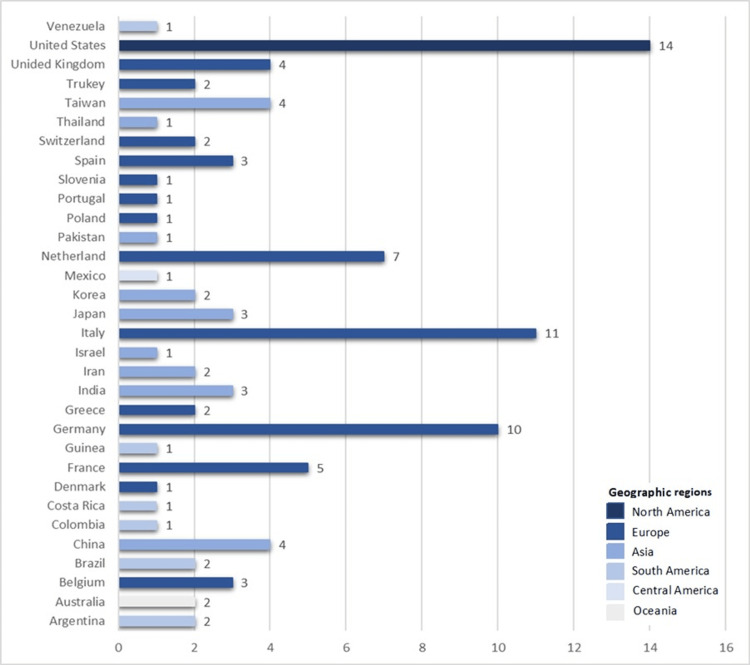
Number of included articles per country and geographic distribution This figure demonstrates the number of included articles per country among the 96 selected articles that meet our criteria. The different colors highlight the geographic regions where the studies were conducted.

**Figure 5:**
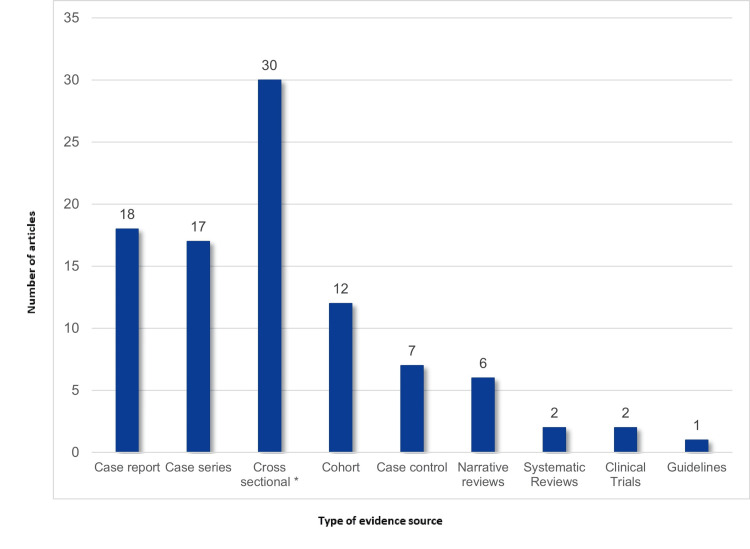
Included types of study design This figure shows the frequency of each included study design among our 96 selected articles. *Screening studies were also included in this category.

Among the 96 selected articles, 83 (86%) were published between 2017 and 2019 (Figure 2). Only three articles were available in Spanish. Most studies were conducted in the United States (14.6%, n = 14), Italy (11.5%, n = 11), Germany (10.4%, n = 10), and The Netherlands (7.2%, n = 7). Four studies examined patients from multiple countries: one assessed patients from Latin America and three from Europe (Supplementary Material 2). In terms of regional representation, studies from South America, Oceania, and Central America accounted for only 8.3% (n = 8), 2% (n = 2), and 1% (n = 1) of selected articles, respectively (Figure 3 and Figure 4). This geographical distribution may reflect disparities in research resources and infrastructure for studying PD, including access to epidemiological data and reliable data registries, diagnostic testing, and funding availability. Cross-sectional studies (31%, n = 30) were the most prevalent study design, while guidelines were the least frequent (1%, n = 1) (Figure 5).

Characteristics of the studied population

Fifty percent of selected articles focused exclusively on LOPD (n = 48), while 22% (n = 21) focused on IOPD. Articles reporting the infantile phenotype used various terms, including IODP (n = 18), prenatal PD (n = 1), and classic infantile PD (n = 2). Twenty-eight percent (n = 27) of the studies simultaneously focused on IOPD and LOPD. Supplementary Material 2 provides additional details regarding inclusion criteria, number of patients, sex distribution, and estimated age per included article.

Assessment methods

Screening

Of the 96 selected articles, 11% (n = 11) described newborn screening (NBS) for PD. Among these, three studies were conducted in North America, three in Europe, and two in Asia. Only one study was from a Central American country, specifically Mexico. Although this distribution indicates some degree of global representation, it underscores significant regional disparities in implementing NBS programs for PD.

Four studies highlighted the role of NBS programs in promoting early diagnosis and treatment, particularly for IOPD. Additionally, six articles emphasized the capability of NBS programs to detect various lysosomal diseases, including PD. One article provided insights into appropriate patient monitoring and the frequency and type of follow-up assessments for all patients identified through NBS (Supplementary Material 2) [24]. While implementing NBS was argued to enhance the accuracy of PD prevalence estimation, other studies stressed the importance of closely monitoring asymptomatic patients with abnormal NBS results [25-30]. In regions with non-existent, incomplete, or inconsistent NBS coverage, affected infants may face delays in screening and subsequent treatment initiation, potentially leading to disease progression. Therefore, equitable NBS implementation can yield better outcomes for affected individuals [29,31-32].

Clinical Presentation

Overall, 84% (n = 81) of references assessed patients' clinical manifestations. Most articles focused on describing clinical manifestations of LOPD (n = 47), as individuals with this phenotype tend to have higher survival rates than IOPD. Furthermore, 16 articles reported manifestations of IOPD, and 18 articles discussed the clinical presentations of both PD subtypes. One article targeted genetic variants and used standardized terms and identification numbers from the Human Phenotype Ontology (HPO) to describe PD phenotypes. Table 2 shows the reported clinical manifestations among articles.

**Table 2:** Reported clinical manifestations for infantile-onset Pompe disease (IOPD) and late-onset Pompe disease (LOPD)

Pompe disease phenotype	Reported clinical manifestation	No. of articles
Infantile-onset Pompe disease (IOPD)	Growth	
	Low body weight	2
	Delay in development	1
	Head and neck	
	Macroglossia	2
	Weakness and inability to control neck muscles	2
	Dysarthria with nasal speech	1
	Enlarged lips	1
	Feeble cry	1
	Swelling of gingiva	1
	Eyes	
	Impaired vision	1
	Multi-directional nystagmus	1
	Ptosis	1
	Ears	
	Hearing impairment	1
	Cardiovascular system	
	Hypertrophic cardiomyopathy/severe infantile hypertrophic cardiomyopathy	7
	Cardiomegaly	2
	Cardiomyopathy	2
	Arrhythmias (e.g., no sustained and sustained supraventricular tachycardia, atrial or ventricular premature beats, and ventricular tachycardia or fibrillation)	1
	Cardiac failure	1
	Cardiac murmur	1
	Dilated cardiomyopathy (e.g., right atrial enlargement and biventricular hypertrophy)	1
	Left ventricular thrombus based at the mitral valve	1
	Mild ventricular dysfunction	1
	Outflow tract obstruction	1
	Reduced ejection fraction	1
	Small ventricular lumen	1
	Systolic bruit	1
	Respiratory system	
	Respiratory distress	4
	Dyspnea	2
	Recurrent airway infections/recurring respiratory infections	2
	Respiratory failure	2
	Tachypnoea	2
	Inspiratory and expiratory muscle weakness	1
	Obstructive sleep apnea	1
	Severe pneumonia	1
	Digestive system	
	Feeding difficulties in infancy, including breastfeeding	3
	Gastric reflux	1
	Genitourinary system	
	Urinary tract infection	1
	Muscles and limbs	
	Hypotonia (including generalized, axial, and proximal)	7
	Muscular weakness (including generalized and distal)	4
	Motor retardation and paucity of movements	2
	Hyporeflexia or areflexia (e.g., decreased or absent deep tendon reflex)	1
	Nervous system	
	Extra-axial cerebrospinal fluid accumulation	1
	Gross and fine motor delay	1
	Delays in speech and social skills	1
	Ventricular enlargement	1
	Unsteady gait	1
	White matter abnormalities (e.g., supratentorial, infratentorial)	1
	Miscellaneous	
	Profound sweating	1
Late-onset Pompe disease (LOPD)	Head and neck	
	Mild to severe neck flexion weakness	3
	Chronic headache	1
	Dysphonia	1
	Severe mild facial weakness	1
	Eyes	
	Subnormal visuospatial functioning	1
	Cardiovascular system	
	Systemic hypertension	3
	Episode of syncope	1
	Respiratory system	
	Dyspnea (including at rest, during and after exercise, and other physical efforts)	11
	Respiratory failure/insufficiency	5
	Orthopnea	4
	Apnea (including sleep apnea-hypopnea syndrome)	2
	Impaired lung function	1
	Nighttime hypoventilation	1
	Restrictive ventilatory deficiency	1
	Recurrent respiratory tract infection	1
	Digestive system	
	Dysphagia (including regurgitation, swelling and feeding difficulties)	7
	Diarrhea	3
	Nausea and vomiting	3
	Belly pain	1
	Bowel incontinence	1
	Constipation	1
	Gas and bloating	1
	Gastroesophageal reflux	1
	Primary biliary cirrhosis	1
	Genitourinary system	
	Renal artery fibromuscular dysplasia	1
	Stenosis of the left renal artery	1
	Muscles and limbs	
	Progressive muscle weakness (including axial and proximal, in the upper and lower limbs)	21
	Difficulties in rising from a seated and supine	5
	Gait disturbances (e.g., subtle waddling gait, Trendelenburg gait, myopathic gait)	5
	Myalgia (including fibromyalgia-like, exertional, and lower-extremity myalgia)	5
	Recurrent falls	5
	Scapular winging	5
	Difficulties to run	4
	Generalized fatigue	4
	Progressive difficulty in climbing stairs	4
	Residual muscle weakness (e.g., in trunk, ptosis, myopathic facies, lumbar lordosis)	4
	Lordosis (including lumbar hyperlordosis)	3
	Walking difficulties (e.g., reduced cadence and speed)	3
	Hypotrophy and atrophy (e.g., of shoulder girdle, pelvic girdle, and paravertebral muscles)	2
	Muscle paralysis (e.g., diaphragmatic and tetraplegia)	2
	Respiratory muscles impairment and weakness	2
	Rigid spine/rigid spine syndrome	2
	Scoliosis	2
	Acute low-back pain	1
	Camptocormia of the lumbar muscles	1
	Decreased lower limb muscle strength and power	1
	Distal and proximal myopathy	1
	Early deviations in skeletal muscle posture and movement	1
	Global hyporeflexia	1
	Increased thoracic and pelvic mobility (e.g., increased thoracic sway, hip adduction angles)	1
	Left-sided scapula alate	1
	Muscular exertion intolerance	1
	Muscle aches and cramps	1
	Thoracic hyperkyphosis	1
	Vertebrobasilar dolichoectasia	1
	Nervous system	
	Delay and regression in gross motor skills	3
	Sleep disorders (including hypersomnia)	2
	Acute cerebral stroke	1
	Apathy	1
	Delayed motor development	1

Laboratory Studies as Diagnostic Methods

A total of 25 articles described biochemical, hematological, or biomarker studies (Table 3). Dried blood spot (DBS) was the most frequently mentioned (n = 18). This test can detect both partial and total decreases in GAA enzyme activity [33-36]. Other reported tests included the quantification of serum creatinine kinase (sCK; n = 17), alanine aminotransferase (ALT; n = 6), aspartate aminotransferase (AST; n = 5), and urinary glucose tetrasaccharide (Glc4; n = 5) (Table 3). These measurements showed elevated levels in PD patients. In particular, muscle-specific enzymes, such as CK and AST, were associated with muscle tissue damage or dysfunction. One article measured serum platelet-derived growth factor-BB (PDGF-BB) to indicate dysregulation of muscle regeneration in PD. This article suggested using PDGF-BB to monitor asymptomatic LOPD patients [37].

**Table 3:** Laboratory and genetic methods to diagnose and monitor Pompe disease

Category	Assessment method	No. of articles	Main findings	Purpose
Hematological, biochemical	Acid alpha-glucosidase activity assay (GAA) in dried blood spot (DBS) leukocytes or lymphocytes, skin or muscle	19	The enzyme activity is often decreased. Quantitative thresholds vary depending on analyzed cells and the profile of assessed patients.	Diagnosis
	Creatinine kinase, including serum CK (sCK) and creatine phosphokinase (CPK)	17	Articles reported elevated levels of sCK, indicating muscle damage. This finding is not exclusive to Pompe disease, and it may occur in other neuromuscular disorders.	Diagnosis and follow-up
	Aspartate transaminase (AST) and Alanine transaminase (ALT)	6	Increased serum AST and ALT levels were documented.	Diagnosis
	Blood gas analysis	6	Blood gas analysis varied with PD severity and impairment of respiratory and metabolic functions. Findings included acidosis owing to glycogen accumulation, hypoxia, and elevated PCO2 due to weakness in respiratory muscles and inefficient exhalation, increased lactate, and altered electrolyte levels (potassium, sodium, chloride).	Diagnosis
	Urinary glucose tetrasaccharide (Glc4)	6	Increased Glc4 levels were documented.	Diagnosis and follow-up
	Serum growth factors, i.e., transforming growth factor β1, platelet-derived growth factor BB (PDGF-BB), platelet-derived growth factor AA, and connective tissue growth factor	2	Symptomatic adult Pompe disease patients presented lower serum concentration of PDGF-BB. This finding might indicate dysregulation of muscle regeneration in Pompe disease.	Diagnosis and follow-up
	Anti-rhGAA antibodies	1	Elevated in response to commencing recombinant human acid alpha-glucosidase (rhGAA) therapy.	Follow-up
	Lactate dehydrogenase (LDH)	1	In some cases, serum LDH levels may be elevated.	Follow-up
	Lysosomal deposit in leukocytes	1	Articles demonstrated the lysosomal deposit of glycogen into various tissues, given the multisystemic nature of Pompe disease.	Diagnosis
Histopathology or immunochemical-based studies	Muscle biopsy	6	Biopsies revealed an excessive accumulation of glycogen within muscle cells. Other findings comprised vacuole formation, atrophic muscle fibers, and signs of myopathy.	Diagnosis
	Muscle biopsy with fluorometric determination of enzyme activity	1	This method helped confirm the diagnosis of Pompe disease.	Diagnosis
Genetic studies	Mutation analysis of GAA by PCR and direct sequencing (Sanger)	7	These methods were used to identify variants in homozygosis or compound heterozygosis. Occasionally, Sanger sequencing could not identify second mutated alleles.	Diagnosis
	Mutation analysis by real-time PCR	1		Diagnosis
	Mutation analysis does not specify the methods	6		Diagnosis
	Next-generation sequencing (NGS)	2		Diagnosis

Genetic Studies as Confirmatory Diagnostic Test

Of 96 included articles, 21 (22%) provided information on genetic variants within the GAA gene. Most articles mentioned Sanger sequencing as the gold standard technique (n = 8; 38%) for detecting gene sequence variants. Three references employed a combination of sequencing methods, including next-generation sequencing (NGS) followed by Sanger sequencing [33,36-37]. Only one paper mentioned the use of NGS deep sequencing as a primary technique to ascertain the genetic status of patients. Furthermore, one study discussed the utility of whole-exome sequencing [38].

All 21 articles referred to databases to assess variant novelty and classified their pathogenicity: very severe, potentially less severe, less severe, potentially mild, presumably non-pathogenic, non-pathogenic, or of unknown significance. The consulted databases included ClinVar, Erasmus MC University Medical Center, Exome Aggregation Consortium, Genome Aggregation Database, Leiden Open Variation Database, and Human Genome Mutation Database. Supplementary Material 3 provides an overview of the extracted genetic information.

Across the 21 articles, several variants were detected, with nonsense mutation being the most prevalent, followed by small deletions, including some in-frame deletions. Other variants included deletions, small insertions, and deletions (indels), and complex rearrangements. The most severe variants affected the catalytic domain of the GAA enzyme (exons 7-15) [38].

Imaging Studies

In total, 44 articles addressed imaging studies (Table 4). Magnetic resonance imaging (MRI) was the most used (n = 31; 70%), assessing the brain, muscles, heart, thoracic region, kidneys, and other anatomical areas. MRI was primarily applied to predict prognosis in LOPD, as it can evaluate the extent of fatty infiltration in muscles and correlate with disease severity. Ultrasound imaging was employed in 30% (n = 13) of the articles, mainly assessing muscles, cardiovascular and respiratory structures. It was often used during initial investigation, detecting characteristic patterns of symmetric fatty infiltration with preserved muscle volume. Cardiac ultrasound frequently revealed left ventricular hypertrophy, aiding in diagnosing and monitoring cardiac involvement. Only 11% (n = 5) and 9% (n = 4) of articles mentioned radiographic studies and computed tomography (CT) scans, respectively. Radiographic studies primarily identified skeletal abnormalities, while CT scans assessed structural abnormalities, such as diaphragmatic elevation and hepatomegaly.

**Table 4:** Imaging methods to diagnose and monitor Pompe disease

Category	Assessment method	No. of articles	Main findings	Purpose
Magnetic resonance imaging (MRI)				
					a) Brain	Brain MRI	5	Patients with PD have an increased risk of intracerebral arterial aneurysms and other vascular defects. Approximately 3% of LOPD patients die from cerebral aneurysmal rupture. Baseline cerebral magnetic resonance angiography is recommended for all patients.	Diagnosis and follow-up
Brain time-of-flight MRI angiography	1	Follow-up						
Magnetic resonance angiography (MRA)	2	Incidence of intracranial dilatative arteriopathy in LOPD is higher than in the general population. No intracranial aneurysms, microbleeds, or significant cerebrovascular disease was found. Abnormalities in the anterior and the posterior circle of Willis correlated with age and disease duration but not with the severity of muscle/respiratory involvement or with genetic data.	Diagnosis and follow-up		
b) Muscle	Muscle MRI	6	Muscle atrophy, fatty infiltration, and occasional hypertrophy of certain muscles can occur.	
	Quantitative muscle MRI	1	The fat content of thigh muscles increased by 1.7% in symptomatic LOPD patients. Similarly, no significant changes were observed in muscle MRI in asymptomatic patients over the year.	Follow-up
c) Heart	Cardiac magnetic resonance	1	Hypertrophy of the left ventricle and alterations in myocardial strain were observed.	Follow-up
d) Thorax	Dynamic kinematic thoracic MRI	1	LOPD patients exhibited significantly lower static and dynamic respiratory function. PIMAX, spirometry, endurance time, and maximal diaphragm descent show significant correlations. During single-breath inspiratory loads, inspiratory time and airflow acceleration increased to preserve volume, with response magnitudes in LOPD correlating to maximal chest wall kinematics. Even in early-stage PD, diaphragm motion was already reduced, and its shape was more curved during inspiration. MRI detected early signs of diaphragmatic weakness in PD patients, aiding in selecting individuals for early intervention to prevent diaphragm damage.	Follow-up
e) Renal	Renal arteriography	1	A 42-year-old man was diagnosed with LOPD after experiencing a renal infarct due to renal artery fibromuscular dysplasia. The association of LOPD and arteriopathy should always be considered in clinical practice.	Diagnosis
f) Others	Whole-body MRI	2	It was used to assess the severity of muscle disease and to monitor LOPD patients over time by measuring the degree of fatty infiltration in LOPD.	Diagnosis and follow-up
	Semi-quantitative evaluation of imaging data (the Mercuri score)	1	Quantifying fatty muscle degeneration using a semi-automated method provided a more detailed overview of disease progression compared to the semi-quantitative Mercuri scoring.	Follow-up
	Quantitative semi-automated evaluation	1		
Computed tomography (CT) scan				
a) Brain	Brain CT	1	Atrophy or changes in brain structure can occur. Mild abnormalities on brain imaging in untreated or newly treated patients with infantile PD tend to resolve over time. Emerging matter changes included abnormal periventricular white matter changes with subtle signal abnormalities in the basal ganglia and minimal, symmetric signal abnormalities involving the deep frontoparietal cerebral white matter. Neuroimaging should be considered as part of the clinical evaluation of IPD to assess for white matter changes and cerebral aneurysms.	Diagnosis
b) Abdomen	Abdominal CT scan	1	It was used to diagnose and monitor muscle involvement.	Diagnosis and follow-up
Radiography (X-ray)				
a) Thorax	Thoracic X-ray	4	It assessed the impact on the respiratory system, including signs like diaphragm elevation and changes in lung volumes. In IOPD, it revealed massive cardiomegaly.	Diagnosis
b) Spine	Spine X-ray	1	It helped assess the impact of skeletal abnormalities in PD.	Diagnosis
Ultrasound				
a) Heart	Echocardiogram	7	Echocardiogram was used to assess and monitor cardiac function, evaluating the size and function of the heart chambers and the movement of the heart's walls. Hypertrophic cardiomyopathy was the most prevalent cardiac manifestation of PD.	Diagnosis and follow-up
b) Respiratory system	Diaphragm ultrasound	2	In LOPD patients, this imaging study showed signs of diaphragm thickening or weakness. Ultrasound studies of diaphragm thickness at functional residual capacity (FRC) correlated with maximal inspiratory pressure (MIP) and seated forced vital capacity (FVC). Diaphragm thickness at total lung capacity (TLC) correlated with MIP and FVC in both seated and supine positions. The thickness of the diaphragm (TF) correlated with MIP and FVC in both seated and supine positions. Notably, diaphragm thickness at FRC correlated with disease duration in LOPD patients. Diaphragm mobility correlated with diaphragm thickness at TLC, FRC, and TF. Additionally, diaphragm mobility correlated with FVC in seated and supine positions and with MIP.	Diagnosis
	Pulmonary sonography	2	It evaluated the diaphragm and other respiratory structures, providing valuable information for managing respiratory complications in individuals with PD. It revealed signs of respiratory distress, such as decreased lung expansion or abnormal diaphragm movement.	Diagnosis
d) Urinary system	Pre/post-micturition bladder ultrasonography	1	It revealed urinary incontinence in LOPD.	Follow-up
e) Others	Echo heterogeneity index	1	The echo heterogeneity index quantitatively described muscle involvement in PD. Lower echo heterogeneity indices in lower limb muscles were associated with worse motor functions in these patients.	Follow-up

Electrophysiological and Functional Studies

A total of 35 articles mentioned electrophysiological and functional studies in the primary clinical evaluation of patients with PD (Table 5 and Table 6). Functional studies were the most frequently cited (89%, n = 31) among the articles devoted to phenotypic assessment, focusing primarily on muscular (35%, n = 11) and respiratory (45%, n = 14) evaluations.

**Table 5:** Electrophysiological studies to diagnose and monitor Pompe disease

Category	Assessment method	No. of articles	Main findings	Purpose
a) Muscles	Electromyography	5	It showed patterns compatible with myopathy.	Diagnosis and follow-up
	Diaphragm nerve conduction study	1	Reduced compound muscle action potential amplitudes and impaired nerve conduction in the diaphragm muscle indicated deteriorating diaphragmatic function.	Diagnosis
	Magnetic stimulation of the diaphragm and abdominal muscles	1	This method allowed for Improvements in respiratory muscle function, enhancing the quality of life and respiratory health of affected individuals.	Follow-up
	Quantitative electromyography (QEMG)	1	QEMG evaluated the extent of muscle involvement and disease severity. Common signs of myopathy included short-duration, low-amplitude motor unit action potentials and increased spontaneous activity at rest.	Diagnosis
b) Heart	Electrocardiogram	5	In IOPD, cardiac conduction defects in long-term survivors included supraventricular tachycardias and Wolff-Parkinson-White syndrome. Other findings comprised a shortened PR interval, an increased QT dispersion (QTd), large left ventricular (LV) voltages, and arrhythmias. Additionally, patients with LOPD are at risk of abnormal cardiac rhythms, short PR interval, and repolarization abnormalities.	Diagnosis and follow-up
	24-hour Holter	1	It detected conduction abnormalities.	Follow-up
c) Sleep	Polysomnography	2	Sleep-disordered breathing: obstructive sleep apnea or respiratory-related sleep disturbances. Irregularities and patterns of respiratory disturbances: disruptions, arousals, or other sleep-related issues affecting the patients.	Diagnosis
d) Brain	Electroencephalogram	1	It detected seizure activity in patients with PD.	Follow-up

**Table 6:** Functional studies to diagnose and monitor Pompe disease

Category	Assessment method	No. of articles	Main findings	Purpose
a) Muscles	Six-minute walk test (6MWT)	16	6MWT provided a reliable measure of walking ability and endurance in PD. It tracked changes in physical abilities over time, establishing associations between walking capacity and disease severity markers. The 6MWT is a validated and valuable tool for assessing functional capacity, monitoring disease progression, evaluating therapeutic interventions, and understanding clinical implications.	Diagnosis and follow-up
	10-minute walk test (10MWT)	5	The 10MWT revealed an important level of variability among patients, likely due to factors such as disease severity, age, treatment response, and individual differences. This variability hindered the establishment of a consistent benchmark for improvement. Patients with more advanced stages of PD often showed a decline in walking ability over time.	Follow-up
	Gross Motor Function Measure-88 (GMFM-88)	3	It utilized loss of motor milestones as a standardized measure used to monitor disease progression.	Diagnosis and Follow-up
	The four-step stair climb test	3	A declining performance in the 4-step stair climb test over time reflected disease progression.	Follow-up
	Quick motor-function test	3	It assessed muscle strength, mobility, and motor skills, providing clinicians with data to monitor disease progression and treatment effectiveness. It also helped track muscle weakness and functional limitations and identify specific areas of weakness.	Diagnosis and follow-up
	Bruininks-Oseretsky Test of Motor Proficiency, Second Edition (BOT-2)	1	It assessed motor skill development, functional abilities, and limitations.	Diagnosis
	Cervical magnetic stimulation	1	It allowed early detection and monitoring of respiratory muscle weakness.	Diagnosis and follow-up
	Coin rotation task	1	It evaluated motor function and fine motor skills.	Follow-up
	Fitbit One™ activity tracker	1	It provided measurements for monitoring physical activity in adults.	Follow-up
	Functional Independence Measure (FIM score)	1	It revealed a decline in functional independence over time, highlighting the progressive nature of PD and the need for monitoring respiratory function.	Follow-up
	Five times sit-to-stand test (FTSST), cervical flexion time in a supine position, and one leg stance	1	The FTSST evaluated the impact of PD on lower limb strength and functional abilities. Muscle weakness, including in the neck, affected the ability to perform this movement.	Follow-up
	Gait Profile Score	1	It assessed multiple gait parameters to understand specific gait impairments associated with the disease.	Diagnosis and follow-up
	Hand-held dynamometry	1	It provided an objective measure to assess functional outcomes and daily living activities in patients.	Follow-up
	Mobility outcome measures (step count and peak one-minute activity)	1	It provided precise and continuous measurements to evaluate patients' functional abilities and capacity to perform daily activities.	Follow-up
	Movement Analysis Profile	1	It showed motor impairments, particularly gait patterns, muscle strength, motor function, and overall movement quality.	Diagnosis
	Quantitative muscle testing	1	It evaluated muscle function over time to identify patterns of weakness progression and develop more precise prognostic indicators.	Follow-up
	Respiratory muscle endurance test	1	It demonstrated respiratory muscle weakness and correlated it with disease severity and progression.	Follow-up
	Strength tests of the paraspinal and psoas muscles	1	Weakness in these muscles contributed to postural abnormalities, mobility issues, and compromised spinal stability, affecting daily activities.	Follow-up
b) Respiratory function	Spirometry	12	Progressive muscle weakness in PD led to reduced forced vital capacity (FVC) and forced expiratory volume in 1 second (FEV1), indicating disease progression. A declining FVC prompted the need for interventions such as respiratory muscle training or ventilatory support. Reduced peak expiratory flow (PEF) and low peak cough flow (PCF) further signaled respiratory muscle weakness and potential respiratory insufficiency. Significant reductions in FVC, maximum inspiratory pressure (MIP), maximum expiratory pressure (MEP), diaphragm excursion, diaphragm thickness at functional residual capacity (FRC), total lung capacity (TLC), and thickness fraction (TF) were observed in LOPD patients compared to controls.	Diagnosis and follow-up
	Single-breath alveolar-capillary diffusion of CO	1	It showed reduced CO diffusion capacity, indicating impaired gas exchange. In severe cases, there was a greater impairment in CO diffusion, which may serve as a marker for disease progression.	Diagnosis
	Single-breath ILC	1	This method demonstrated lower static and dynamic respiratory function in LOPD.	Follow-up
	Transcutaneous nocturnal capnometry	1	This method investigated sleep-disordered breathing and nocturnal hypoventilation.	Diagnosis
c) Neurological assessment	Montreal Cognitive Assessment (MoCA) Test	2	It was utilized to assess cognitive impairment	Diagnosis and follow-up
	Rey Auditory Verbal Learning Test	1	It revealed changes or declines in cognitive abilities over time.	Follow-up
	Wisconsin Card Sorting Accepted Article Test	1	It demonstrated that PD patients may experience impairment in cognitive ability, decision-making, problem-solving, and adaptability.	Follow-up

Among electrophysiological studies, electromyography (EMG, n = 5) contributes to the diagnostic stage by detecting brief early recruiting potentials (myotonic discharges), particularly in the paraspinal muscle, and spontaneous activities such as fibrillations and positive sharp waves. While these findings may indicate PD, they can also appear in other neuromuscular disorders, thus highlighting the importance of combining EMG with other diagnostic tests. Electrocardiography (ECG, n = 5) was used to identify cardiac involvement, commonly detecting features such as short PR intervals and preexcitation patterns. Electroencephalogram (EEG, n = 1) was less frequently mentioned but revealed abnormalities in patients with central nervous system involvement.

Scales and Questionnaires

Forty-four studies documented findings from scales and questionnaires. Among the articles, 38 reporting scales are not specific to PD. These generic scales evaluated various domains, including muscle function, respiratory function, gastrointestinal symptoms, cognitive function, and neurological disability. Only two articles employed disease-specific instruments, such as the Pompe Disease Symptom Scale (PDSS) and Pompe Disease Impact Scales (PDISS), which assess symptom severity and overall impact on the daily life of PD patients, respectively. Four articles cited questionnaires to evaluate pain, neuromuscular symptoms, and respiratory symptoms (Table 7).

**Table 7:** Scales and questionnaires to diagnose and monitor Pompe disease

Category	Assessment method	No. of articles	Main findings	Purpose
Non-specific scales for PD				
a) Muscles	Modified Medical Research Council (MRC) grading scale	5	It associated these scores with clinical outcomes to assess the severity of respiratory muscle weakness.	Diagnosis and follow-up
	Muscle Research Council scale (MRC)	4	It contributed to monitoring muscle strength over time and assessing disease progression.	Follow-up
	Activity limitations (ACTIVLIM) scale	2	It monitored muscle strength over time, assessing disease progression.	Follow-up
	Alberta Infant Motor Scale (AIMS)	2	It helped detect developmental delays, including motor skill impairments.	Diagnosis
	Motor function measure-20 item scale (MFM-20)	2	It showed changes in motor abilities over time.	Follow-up
	Peabody Developmental Motor Scales - Second Edition (PDMS-2)	2	It assessed motor abilities in children.	Diagnosis
	Short Form (36) Health Survey (SF-36) scale scores	2	This scale showed that physical, emotional, and social aspects of PD patients' health were impacted.	Follow-up
	Gardner-Medwin Scale	1	The scale assesses muscle strength and function across various muscle groups, including neck flexors, neck extensors, shoulder abductors, elbow flexors, and hip flexors. It helps track disease progression and treatment response in PD patients.	Diagnosis and follow-up
	Gowers-Walton Scale	1	It assessed how proximal muscle weakness affects muscles closer to the trunk, such as those in the shoulders, hips, and thighs. Such a manifestation can lead to motor function impairments, especially in tasks like standing up from a seated position, walking, and climbing stairs. Functional abilities can range from mild to severe impairment.	Diagnosis
	Mercuri grading scale	1	It evaluated the extent and severity of muscle involvement and weakness.	Diagnosis
	Rotterdam Handicap Scale	1	It assessed disabilities in daily life activities, participation, and social interaction.	Follow-up
	Walton and Gardner-Medwin (WGM) scale	1	It evaluated the impact of the disease on muscle strength, identified areas of weakness, and monitored the effectiveness of treatments or interventions.	Diagnosis and follow-up
b) Neuropsychiatric	Wechsler Intelligence Scale third edition for children (6-16 years old)	2	Findings vary depending on disease progression and impact on neurological functioning. The cognitive profile encompasses various domains, such as processing speed, executive functions, and working memory.	Diagnosis and follow-up
	Wechsler Adult Intelligence Scale fourth edition (>16 years)	1		
	Epworth Sleepiness Scale (ESS)	1	Excessive daytime sleepiness is more prevalent in PD patients compared to the general population, leading to a lower quality of life.	Diagnosis
	Hospital Anxiety and Depression Scale (HADS-D)	1	This scale showed increased levels of psychological distress in PD patients due to factors such as the burden of managing the disease, limitations in daily activities, uncertainty about the future, and the impact on social and emotional aspects of life.	Follow-up
c) Respiratory	Medical Research Council Dyspnea Scale	2	It assessed the severity of respiratory muscle weakness and its impact on breathing difficulties. There was a correlation between MRC dyspnea scale scores and the severity of PD.	Diagnosis
d) Gastrointestinal	Patient-Reported Outcomes Measurements Information System - Gastrointestinal (PROMIS-GI) Symptom Scales	1	It assessed gastrointestinal symptoms as part of the overall symptom profile.	Diagnosis
e) Others	Pain Severity Score Scale/Brief Pain Inventory Questionnaire	2	PD patients obtained high score values with this scale, showing how pain interferes with their general activities such as walking, work, mood, sleep, and enjoyment of life.	Diagnosis and follow-up
	Fatigue Severity Scale (FSS)	1	Fatigue was highly prevalent among both mildly and severely affected adult patients with PD, who obtained high mean scores on this scale.	Diagnosis and follow-up
Specialized scales				
	Pompe Disease Symptom Scale (PDSS)	1	This scale provided a more nuanced understanding of symptom variability among age groups and phenotypes.	Follow-up
	Pompe Disease Impact Scale (PDIS)	1	This scale assessed the impact of PD in daily living, respiratory function, muscle strength, fatigue, and emotional well-being.	Follow-up
Questionnaires				
a) Muscles	Individualized Neuromuscular Quality of Life (INQoL) questionnaire	2	Findings from this scale demonstrated that PD patients experience limitations in physical activities due to muscle weakness and fatigue. Anxiety, depression, and overall emotional well-being were also affected by the disease. Fatigue and reduced energy levels that impact daily life and activities.	Follow-up
b) Respiratory	Respiratory Symptom Questionnaire (RSQ)	1	It identified respiratory muscle weakness and gradual decline in respiratory function as prominent manifestations in individuals with PD. This resulted in breathing difficulties and a consequent impact on daily activities and social interactions.	Diagnosis and follow-up

The psychometric properties of reported scales and questionnaires, such as reliability and validity, varied significantly. Disease-specific scales like PDSS and PDIS have demonstrated sensitivity to changes in PD progression and treatment responses. Nonetheless, such specialized scales may be applied less often than generic scales. In contrast, generic scales enable the comparison of health outcomes across different conditions but may overlook disease-specific nuances, such as muscle strength or respiratory function. Understanding these differences can elucidate the applicability and limitations of scales in assessing PD-related symptoms and functional impairment over time.

Follow-Up of Patients With Pompe Disease

Functional assessments emerged as the predominant diagnostic tools in PD follow-up. These assessments encompass a range of parameters, including muscle strength evaluations and respiratory function, with spirometry being the most frequently mentioned (n = 52). Articles also reported pulmonary function test (PFT) and polysomnography (n = 2) to monitor respiratory distress.

Regularly evaluating swallow function was crucial for assessing oropharyngeal muscle weakness in IOPD and progressive LOPD. In particular, articles studies recommended serial assessments, including video-fluoroscopic swallow studies (VFSS), to monitor swallowing function effectively [39,40].

Additionally, reviewed references reported that functional assessments for LOPD should comprise muscle strength measurements, comprehensive neuromuscular stratification, and metabolic monitoring using quantitative muscle MRI (T1-weighted imaging) or the Dixon scale. Qualitative assessments need to cover posture, movement, and musculoskeletal status. Standardized tests, such as the Alberta Infant Motor Scale (AIMS, n = 2) and Gross Motor Function Measure (GMFM, n = 3), should also be considered. Follow-up protocols should incorporate MRI as this imaging technique helps detect structural muscle changes in symptomatic patients. Furthermore, we found that only one article mentioned monitoring methods that evaluated patient-reported outcomes measures (PROMs), such as the Impact of Neuromuscular Diseases on Quality of Life (INQoL) [41].

Discussion

Our study is the first to comprehensively map and characterize methods for PD screening, diagnosis, and monitoring. We reviewed 96 articles from 32 countries, mainly Europe and North America. Articles on PD screening highlighted the role of NBS programs in early disease detection to improve patient outcomes and prevent irreversible complications. We identified various diagnostic tools, including enzyme assays and genetic testing, each with its own strengths and limitations. Enzyme assays, such as DBS tests, are commonly used for initial diagnostic assessment due to their accessibility and cost-effectiveness, whereas genetic testing, though more resource-intensive, provides a definitive diagnosis. We noted significant heterogeneity in classifying PD's clinical spectrum, complicating the standardization of diagnosis and follow-up. Even though imaging techniques and functional assessments are essential for monitoring disease progression, a lack of consensus on monitoring protocols hampers their application in clinical practice. These findings underscore the need for a multifaceted approach to PD healthcare, incorporating diverse methods for timely diagnosis and high-quality follow-up care.

Screening

NBS programs have been established in numerous countries to facilitate the early detection of PD [25,27,42-44]. However, NBS programs are not universally available [27]. The included articles showed disparities in NBS implementation, with some countries having pilot programs while others are in the early planning phase [45-49]. Such disparities may occur owing to national differences in health systems' infrastructure, resources, and governmental priorities [50-52].

Regarding the sensitivity and specificity of NBS compared to other screening methods, NBS can become the primary screening tool due to its ability to detect affected individuals before the symptom's onset. The data obtained from NBS are also instrumental in understanding PD's prevalence and differentiating it from other musculoskeletal disorders [28,53]. However, NBS has limitations, including the potential for false positives and negatives. Despite constraints, cost-effectiveness analysis of NBS programs for PD has demonstrated their potential to reduce long-term healthcare costs by enabling early detection and intervention, which can prevent disease complications associated with PD. The cost-effectiveness of NBS programs must be evaluated within the context of each country's healthcare system, considering factors such as screening program cost, treatment expenses, and resource utilization. By identifying barriers and facilitators to program adoption and sustainability, stakeholders can develop targeted strategies to enhance the NBS program's effectiveness and maximize its impact on patient outcomes.

Diagnostic Biochemical and Genetic Methods

Biochemical assessments are indispensable for detecting and monitoring PD's progression [54-57]. In our review, DBS was reported in 18 articles and emerged as the predominant method among reviewed enzyme assays. DBS can detect partial and total decreases in GAA enzyme activity. However, other pseudodeficiencies or altered expression of GAA enzyme that do not result in PD should be considered when interpreting the test results [54]. By characterizing the widespread use of DBS, our review emphasizes this method's potential feasibility and reliability in diagnosing PD, including in countries with limited resources [26] since PD has available and under-developed therapeutic options [58-60].

Nevertheless, we observed a lack of standardization in reporting DBS results, particularly in specifying quantitative thresholds. For example, eight articles characterized GAA deficiency solely through qualitative terms like "reduced," "decreased," "significantly decreased," "low limit level," and "deficient activity," whereas seven articles omitted such descriptive qualifiers. This variation in reporting GAA activity highlights the need for standardization in conveying these results to foster further research correlations, enhance applicability to clinical practice, and facilitate its implementation [54].

As a monogenic recessive disease, diagnosing PD requires the identification of at least two mutations in the GAA gene. In cases where a single or no mutation is detected, diagnosis relies on reduced GAA activity in at least two tissues, with blood and skeletal muscle being the most frequently examined [61]. This approach follows the recommendations of the European Pompe Consortium [35]. In our review, most selected papers emphasized the need for confirmatory genetic analysis of the GAA gene following biochemical observation of reduced GAA activity [5,36,62-64]. Some articles highlighted the limitations of traditional Sanger sequencing in detecting the two alleles [2,31,65-67]. Notably, seven of the 21 articles that applied genetic methods did not specify the sequencing strategy they utilized. However, studies utilizing NGS as a diagnostic tool did not encounter these difficulties [31,36,66,68]. Based on the articles we included, NGS should be considered the gold standard for accurate patient genotyping [31], while Sanger sequencing should be reserved for mutation confirmation when necessary.

While "classic infantile" PD patients have mutations that disrupt all forms of the GAA protein, the precise relationship between specific genotype and phenotype expressions remains elusive [2,3,5]. In this context, the cross-reactive immunologic material (CRIM) status is relevant to classify IODP into two groups: those with detectable (CRIM+) and undetectable GAA protein (CRIM-). The latter group demonstrates a worse prognosis and response to enzyme replacement therapy [69]. The CRIM status can be determined through genotype predictions or a rapid turnaround blood-based leukocyte assay [70,71]. In our review, only one article reported the CRIM status of each evaluated patient [68]. Further research and comprehensive reporting are warranted to elucidate the interplay between genotype, phenotype, CRIM status, and varying levels of GAA enzyme activity.

Although some articles identified new gene variants, these variants were unavailable on the PD reference database [4]. This finding highlights the urgent demand for creating a universal database dedicated to PD-related variants, which should serve as a point of reference [3,4,38]. Such initiatives are essential to standardize the documentation of genetic mutations using widely accepted molecular nomenclature.

Evaluation of Clinical Manifestations

A pervasive challenge among reviewed articles was the heterogeneous classification of PD's clinical spectrum [2,72]. In this regard, the classification proposed by Güngör and Rouser offers an alternative that could be universally adopted for categorizing PD subtypes [73], given its several advantages. This classification system considers both clinical and genetic factors, allowing for a more comprehensive understanding of the disease's heterogeneity. The classification by Güngör and Rouser delineates three yet overlapping categories within PD's clinical spectrum: (1) "classic infantile" PD (CIPD), diagnosed within the first year of life and representing the severe form of PD; (2) paediatric PD (POPD), encompassing patients with clinical onset from birth to adolescence (e.g., ≤14 years) but without persistent and progressive cardiac hypertrophy; and (3) adult PD (AOPD) for patients with symptom onset from adolescence (e.g., ≥14 years) to late adulthood. Achieving consensus in reporting is crucial, as it aids healthcare professionals in increasing awareness of PD clinical manifestations in infants. In addition, the lack of standardized terminology and diagnostic criteria exacerbates the difficulty in identifying patients for clinical trials and other research initiatives. Divergent classification can result in the inclusion of heterogeneous patient populations in studies, confounding research results and impacting their generalizability. By categorizing patients based on clinical presentation, age of onset, and genetic mutations, Güngör and Rouser's classification system provides a framework for researchers and clinicians to identify common patterns within different PD subgroups, aiding in the development of targeted therapies and personalized medicine approaches.

While the clinical manifestations described in the selected papers provided characteristic indicators that raise suspicion of PD, only one article explicitly described observed symptoms using standardized terms and identification numbers proposed by HPO [38]. As current literature exhibits significant discrepancies in clinical reporting of patient phenotypes, promoting a standardized approach can facilitate clarity and consistency in phenotype descriptions, enabling comparison across publications.

Follow-Up

After confirming the diagnosis, individuals with PD require long-term follow-up. The frequency of follow-up visits may vary according to individual needs and disease progression. Our review identified a need for more consensus regarding the best approach to monitor LOPD cases. Moreover, existing guidelines on PD follow-up were not consistently adhered to [74]. Implementing the recommended follow-up guidelines for PD patients presents several challenges, which may hinder the provision of optimal care.

Limited resources, including healthcare infrastructure and funding, are significant barriers to establishing and maintaining comprehensive follow-up programs. Furthermore, variability in healthcare professionals' knowledge and adherence to guidelines can lead to inconsistencies in clinical practices and gaps in health assistance. Geographical accessibility to health facilities, socio-economic status, and cultural beliefs may also influence patients' adherence to recommended proposed treatments. To overcome these barriers, collaborative strategies involving multidisciplinary healthcare teams are essential. For instance, collaboration among specialists in neuromuscular diseases, cardiology, pulmonology, and psychology promotes comprehensive care that addresses both the physical and psychological aspects of PD.

The knowledge of the diagnosis, particularly in individuals without symptoms or functional impairment, may trigger the development of psychological conditions that impact their QOL, such as anxiety or depression [75-77]. Patients and families may benefit from psychosocial support to cope with the emotional and psychological distress associated with this chronic and progressive condition [78,79]. As observed in this scoping review, there is limited focus on the QOL of LOPD patients, with only one paper mentioning the use of INQOL as a PROM [41]. Establishing a national disease registry would provide a starting point for administering QOL assessments among PD patients and increase the number of related studies [75,76]. Additionally, integrated care models with psychosocial support programs have been shown to positively improve patient outcomes and QOL. These programs may include individual or group counselling, peer support networks, educational workshops, and access to community resources. By addressing the comprehensive needs of PD patients, including their psychological well-being, integrated care models can enhance overall patient satisfaction, adherence to treatment plans, and long-term health outcomes [80,81].

In addition to clinical evaluation, scales and questionnaires play a crucial role in assessing PROMS and the impact of PD on patients' lives [42-44]. These tools provide insights into functional limitations, symptom severity, and the overall QOL experienced by PD patients [45]. However, our review highlighted a predominant use of generic scales not specific to PD, with a minority employing disease-specific instruments like the PDSS and PDIS [46]. The utilization of disease-specific PROMs can enhance the accuracy and relevance of clinical assessments by capturing disease-specific nuances that generic scales may overlook [47]. Future research should focus on validating and standardizing these instruments across different cultural and linguistic contexts to facilitate cross-study comparison and improve clinical decision-making in PD management.

Another recognized concern was the need to continuously monitor asymptomatic PD patients identified through NBS. Notably, one study mentioned serum PDGF-BB as a potential biomarker [24]. PDGF-BB levels may indicate dysregulation of muscle regeneration, suggesting its potential use in monitoring asymptomatic LOPD patients. This emerging biomarker warrants further investigation to clarify its role in disease follow-up [24].

Limitations

Despite the comprehensive scope of this review, we must acknowledge our study's limitations and potential biases in the included articles. Our inclusion criteria were limited to articles published in English or Spanish, potentially excluding relevant research published in other languages and introducing language bias. Since we considered articles published up to February 8, 2022, we might have missed recent advancements in PD assessment for clinical practice, leading to a temporal bias. The exclusion of research published after the cut-off date might overlook advancements in PD that are relevant to clinical practice. Although we applied broad eligibility criteria, our review exhibited an overrepresentation of studies from specific geographic regions, introducing regional bias and limiting the application of our findings to other populations. Additionally, variation in study design, sample size, and methodology across the included articles could introduce selection bias and impact the generalizability of findings. We did not include grey literature, which may have excluded relevant evidence and contributed to a selection bias. Our review did not assess the sensitivity and specificity of diagnostic tests, as this was not our focus. Future research could systematically analyse these performance metrics across different assessment methods to provide deeper insights into their relative strengths and limitations. Lastly, we did not evaluate individual study biases since this was beyond the scope of this review. Future research should consider these limitations to improve our understanding of PD assessment.

## Conclusions

Our scoping review comprehensively evaluated reported assessment methods for PD screening, diagnosis, and follow-up. By synthesizing the prevalence and findings associated with these methods, our review provides valuable insights to guide healthcare providers to expedite diagnosis and monitoring, especially in infants carrying common genetic variants. For instance, promoting specific screening methods, such as NBS initiatives, can enhance early detection of PD, improve clinical outcomes, and reduce disease burden. Despite the progress in PD assessment, our review highlighted the need for a more standardized approach, throughout assessment categories. Standardization would not facilitate more accurate diagnoses and also improve the comparability of research findings, ultimately enhancing the quality of healthcare provided to patients. As research in PD continues to evolve, remaining updated on emerging assessment techniques is imperative to ensure the condition's timely and effective management. By leveraging the insights gained from our review, healthcare providers can optimize their approach to PD screening, diagnosis, and monitoring, ultimately improving patient outcomes and QOL.

## Appendices

Supplementary Material 1 

**Table 8:** Selected articles that focused on genetics analysis in Pompe disease For each reference, patient information, the origin of the studied population, and other tools are also detailed. (*) As described in the paper. (**) As indicated in the paper or PD database https://www.pompevariantdatabase.nl/. (***) It was already published in 2016 but not included in the PD database that year. In bold variations are not mentioned in https://www.pompevariantdatabase.nl/, and those highlighted in italics do not respect the nomenclature. CK, creatine kinase; DBS, dried blot spot; EMG, electromyography; ENMG, electroneuromyography; MB, muscular biopsy; MUP, motor unit potentials; NA, not applicable; NBS, newborn screening; NI, not informed; OR, odds ratio; P1, P2…Px, patient one, two…x; PWS, positive sharp waves

Reference	PD phenotype	Patient’s information	CRIM status	GAA activity (*)	Gene variant (*)	Zygosity	Gene variant location	Variant classification (**)	Population/patient origin	Diagnostic method
											Ullah et al., 2020 [34]	IOPD	P1 and P2: From two different families, both patients were born to consanguineous unions. High levels of sugars in the urine and CK in the blood. Myopathy, hypotonia, cardiorespiratory defects, tongue enlargement, failure to gain weight and grow at an expected rate, trouble feeding, slow motor movements, and progressive prostration.	NI	Deficient activity	P1: c.2561G>A; c.2561G>A	Homozygous	18?	Pathogenic?	Pakistani	Sanger sequencing and specific PCR
P2: c.2773A>C; ?	Compound heterozygous?	19?																			
			Tsai et al., 2017 [33]	Classic infantile/IOPD	Infant with prenatal onset of dilated cardiomyopathy who presented at birth with significant cardiorespiratory symptoms. initial chest X-ray showed significant cardiomegaly and mild diffuse pulmonary markings; the ECG showed sinus rhythm with a ventricular right axis deviation without pathognomonic changes of PD; the 2D-echocardiogram (ECHO) demonstrated dilated cardiomyopathy with hypokinesia of left and right ventricles (LV and RV). On examination, the child had tachypnoea and tachycardia with a grade II ejection systolic murmur at the left upper sternal border with intermittent gallop. Extremities were freely moveable. Hypotonia was not obvious. Newborn screening was normal. Negative family history for neuromuscular diseases but significant for multiple sclerosis and seizure disorders. Myocardial biopsy was performed on day of life 30, which revealed marked vacuolization of cardiac myocytes and raised concern for a metabolic disorder. DNA testing by Sanger sequencing revealed no pathogenic variants. CK: 29.6 mmol/mol creatinine (reference range: <19 for age <6 months).	NI	Significant deficiency (4 pmol/hr/spot, reference range 24–94),	c.1441T>C; c.2481+109_c.2646+38del538	Compound heterozygous	10; 18						NI	US resident	NGS deep sequencing and Sanger sequencing			
Li et al., 2018 [82]	Classic infantile	Pregnant women receiving prenatal diagnosis of LSDs. Seven patients suggested with PD four were confirmed.									NI	Proband: 14.16% (% of normal control mean), Fetus: 17.50%	Proband: c.1935C>A; c.2622G>T/Fetus: c.1935C>A; ?	Fetus: compound heterozygous?	Fetus: 14; ?				NI	Chinese	Sanger sequencing
												Proband: 1.22% (% of normal control mean), Fetus: 17.50%	Proband: c.1411-1414del4; c.1935C>A/Fetus: c.1935C>A; ?	Fetus: compound heterozygous?	Fetus: 14; ?						
												Proband: 0.00% (% of normal control mean), Fetus: 33.40%	Proband: c.1437G>C; c.1935C>A/Fetus: c.1437G>C; ?	Fetus: compound heterozygous?	Fetus: 9; ?						
												Proband: 9.80% (% of normal control mean), Fetus: 13.80%	Proband: c.1935C>A;*IVSS20+5G>A*/Fetus: *c.IVSS20+5G*>A; ?	Fetus: compound heterozygous?	Fetus: ?; ?						
Bergsma et al., 2019 [36]	Childhood or adult PD	Patients enrolled in this study were all diagnosed with Pompe disease on the basis of GAA enzyme deﬁciency and the presence of two disease-associated variants in the GAA gene, at least one of which was c.-32-13TNG. One hundred thirty-one compound heterozygous patients enrolled in this study had a conﬁrmed fully deleterious variant on the second allele that did not produce any functional GAA enzymatic activity, as determined on the basis of clinical information. symptomatic patients had skeletal muscle and/or respiratory involvement. Childhood-onset patients are patients younger than 18 years of age at the onset of the disease who do not have hypertrophic cardiomyopathy. Adult-onset patients are patients who are 18 years of age or older at the onset of the disease. (see Tables S2 and S3 for more info).									NI	All patients enrolled had deficient Ez activity	IVS1 patients, the synonymous variant c.510CNT was uniquely present on the IVS1 allele in 9/33 (27%) patients with childhood-onset but was absent from 110 patients with onset in adulthood. GAA enzyme activity was lower in ﬁbroblasts from patients who contained c.510CNT than it was in patients without c.510CNT. By reducing the extent of leaky wild-type splicing, c.510CNT modulated aberrant splicing caused by the IVS1 variant. The deleterious effect of c.510CNT was also found in muscle cells, the main target cells in Pompe disease. In homozygous IVS1 patients, the c.510CNT variant was absent in 4/4 (100%) asymptomatic individuals and present in 3/6 (50%) symptomatic patients. In cells from homozygous IVS1 patients, c.510CNT caused reduced leaky wild-type splicing.	Compound heterozygous or homozygous	All along the gene				NA	Dutch (Netherlander)	Allele-speciﬁc PCR and RT-PCR and long-range allele-speciﬁc PCR and Sanger sequencing
																						Guevara-Campos et al., 2018 [65]	Juvenile-onset PD	P1: 14 years-old boys has progressive establishment of generalized fatigue, and difficulties in performing activities. Cramps and weakness and reduced muscular strength, mainly in limbs, generalized hypotrophy, and loss of strength in his lower limbs. CK: 2770 U/L	NI	Low limit level: 14.10 mmol/L/h (reference 15mmol/L/h)	c.547-67C>G and c.547-39T>G	Homozygous/homozygous	2i	NI	Venezuelan	Sanger sequencing
P2: Seven-year-old boy complaint of easy fatigue while walking and climbing stairs, he fell several times. He presented weakness, reduced muscular strength, and pain in his limbs. CK: 2680 U/L	Decreased: 5.28 mmol/L/h (reference 15mmol/L/h)	c.1064T >C; c.380G >T									Compound heterozygous	6; 2																				
P3: Six-year-old boy with generalized fatigue and difficulties running and doing sports. Weakness and pain in limbs. CK: 1511 U/L	Decreased: 11.09 mmol/L/h (reference 15mmol/L/h)	*c.32-13T>G* (old name IVS1-13T>G); ?									Homozygous?	1bi																				
Rairikar et al., 2017 [83]	LOPD	P1: Within the first week of life: Generalized hypotonia, delayed gross motor skills, bilateral scapular winging dysphagia. Failure to thrive	NI	Low levels by NBS	c.-32-13T>G; *c.525delT*	Compound heterozygous	1bi; 2	NI	US resident	NI
		P2: Since birth: Generalized hypotonia, delayed gross motor skills, dysphagia, positive family history								
		P3: At five months: Generalized hypotonia, delayed gross motor skills, regression in gross motor skills			c.-32-13 T>G; c.2188G>T	Compound heterozygous	1bi; 15			
		P4: Reported to be asymptomatic			c.-32-13T>G; c.-32-13T>G	Homozygous	1bi			
		P5: Age three months: Regurgitation of feeds, especially when lying supine, self-limited gasping episodes								
		P6: Reported to be asymptomatic								
		P7: Reported to be asymptomatic								
Kishnani et al., 2019 [35]	IOPD and LOPD	113 enrollees (60 male/53 female) aged 1-18 years, 87 had infantile-onset Pompe disease (IOPD) and 26 late-onset (LOPD). One hundred eight enrollees with GAA genotypes had 215 pathogenic variants (220 including combinations). (ADVANCE study)	Supplementary Table S5 lists CRIM statuses	Deficient	Genetic data comprising 215 GAA pathogenic variants (220 including in-cis combinations) (Figure 1; Supplementary Table S1). Four IOPD patients and one LOPD had missing genotypes. Two patients had three variants, two in cis and one in trans, and one patient had four variants: two in-cis combination in-tras to each other. For detailed information, refer to the mentioned Supplementary Table in the paper.	Homozygous and compound heterozygous (see Supplementary Table S1 in the paper)	All along the gene	See Supplementary Table S1 in the paper	US resident	NGS only in a few patients who did not have a genetic profile
Chen et al., 2017 [84]	IOPD	Twenty-ﬁve Chinese patients with IOPD were enrolled, and clinical data were retrospectively reviewed. median age at symptom onset was 3.4 months (range: 1.0-7.1 months) and 4.9 months (range: 2.7-8.3 months) at diagnosis.	NI	Reduced activity in leukocytes or DBS	In 25 IOPD patients and identiﬁed 30 different mutations, 17 of which had been previously cited and 13 of which were novel. No novel variants were found among 120 alleles of normal controls, suggesting that they were uncommon polymorphisms. Several mutations were encountered more than once, including the nonsense mutation c.2662G>T. For details, review Tables 1 and 2 in the paper.	Homozygous and compound heterozygous (see Table 1)	All coding exons (e2–e20) and intron/exon boundaries	See Table 3	North and South Chinese pop	Sanger sequencing
Ebbink et al., 2018 [70]	Classic IOPD	Follow up, assessment tools, neuropsychological tests, and brain magnetic resonance imaging (MRI). From approximately age 2 years onwards, brain MRI showed involvement of the periventricular white matter and centrum semi-oval. After 8 years of age, additional white matter abnormalities occurred in the corpus callosum, internal and external capsule, and subcortical areas. From 11 years of age, white-matter abnormalities were also found in the brainstem. Although there seemed to be a characteristic pattern of involvement over time, there were considerable variations between patients, reﬂected by variations in neuropsychological development. Cognitive development ranged from stable and normal to declines that led to intellectual disabilities.	8 positive; 2 negative; 1 unknown	Less than 1%	See Table 1 in the reference	All homozygous?	Coding exons	NA	Dutch	NI
Moravej et al., 2018 [85]	IOPD	Female neonate with hypertrophic cardiomyopathy, which was detected on echocardiography. and a positive family history of IOPD. In physical examination, the muscular tone was normal, the tongue was not enlarged, and the facial picture was normal. Heart examination was normal, and she did not have organomegaly.	NI	DBS: decreased activity	c.730C>T	Homozygous	4	Very severe	Iranian	Sanger sequencing and PCR to screen for the common deletion of exon 18 were also performed.
Semplicini et al., 2018 [86]	LOPD	Data were collected from the two main laboratories involved in PD diagnosis and from the French Pompe registry. Two hundred 46 patients were included, with a mean age at diagnosis of 43 years. The authors characterized the French cohort of LOPD patients through a nationwide study covering more than 40 years. Clinical data, enzymatic test on lympho/leukocytes and confirmation on fibroblasts, or enzymatic test on lympho/leukocytes or enzymatic test on dried-blood spots. Inclusion criteria were as follows: (1) confirmed diagnosis of PD, established with at least two different methods (either enzymatic test on lympho/leukocytes and confirmation on fibroblasts, or enzymatic test on lympho/leukocytes and confirmation by GAA gene sequencing, or enzymatic test on dried blood spots and confirmation by GAA gene sequencing); (2) disease onset ≥ 2 years of age. Some patients had incomplete genetic (i.e., molecular analysis not performed, one unidentified allele, absence of informed consent) or clinical (age at onset, first symptoms) data.	NI	NI	causative mutations were found in 185 patients. In the 19 remaining patients, only one mutation was identified	See Tables S1and S2	spread all over the sequence except exons 16 and 20	83 different pathogenic or probably pathogenic variants	French	Sanger sequencing and specific PCR
											The common c.-32-13T>G mutation was detected in 89% of index cases on at least one allele,	Compound heterozygous
					c.-32-13T>G; c.-32-13T>G (in 3 patients) (allele frequency = 48.1%)	Homozygous						
					Recurrent variants: c.2481+102_2646+31del, (7.5%); c.1927G>A (3.7%) and c.655G>A (2.7%), and the small deletion c.525del (2.4%).	Compound heterozygous					Disease-causing mutations (Alamut® software)
					Other mutations had an allele frequency ≤ 2% (Table 1); c.-32-13T>G	Compound heterozygous					See Table 1
					28 (34%) Novel mutations: Table 2	NI		NI		
					Bekircan-Kurt et al., 2017 [64]	LOPD		P1: Proximal myopathy. CK(U/L): 491. Muscle biopsy (MB): Vacuolar myopathy. ENMG: PSW, ﬁbrillation, myotonic discharges, myopathic MUP			NI	13% (given as percent ratio of the cutoff value of the laboratory studied). Low muscle Ez activity.	c.547-39T>G; c.547-39T>G/c.858+5ins7; c.858+5ins7	Homozygous/homozygous	2i; 4i	NI	Turkish	NI
								P2: Distal and proximal myopathy. CK(U/L): 1600. MB: Vacuolar myopathy. ENMG: PSW, ﬁbrillation, myopathic MUP				70% (idem previous). Low lymphocyte Ez activity.	c.547-39T>G; c.547-39T>G/c.858+5ins7; c.858+5ins8	Homozygous/homozygous	2i; 4i			
P3: Proximal myopathy. CK(U/L): 700. MB: Vacuolar myopathy. ENMG: PSW, ﬁbrillation, myotonic discharges, myopathic MUP	24% (idem previous). Low DBS Ez activity.	IVS1-13T>G (c32-13T>G); E888X 2662G>T	Compound heterozygous	1bi; 18														
P4: Proximal myopathy, rigid spine. CK(U/L): 602. MB: Vacuolar myopathy. ENMG: PSW, ﬁbrillation, myotonic discharges, myopathic MUP	19% (idem previous). Low DBS Ez activity.	Novel mutation: c.1209C>A; *IVS1-13T>G (c32-13T>G)*	Compound heterozygous	8;1bi														
P5: Proximal myopathy, rigid spine. CK(U/L): 933. MB: Vacuolar myopathy. ENMG: Myotonic discharges, myopathic MUP	15% (idem previous). Low DBS Ez activity.	*IVS1-13T>G*; 2737dupG	Compound heterozygous	1bi; 18?														
P6: Proximal myopathy. CK(U/L): 1588. MB: Vacuolar myopathy. ENMG: PSW, ﬁbrillation, myotonic discharges, myopathic MUP	18% (idem previous). Low DBS Ez activity.	IVS1-13T>G (c32-13T>G); IVS1-13T>G (c32-13T>G)	Homozygous	1bi														
P7: Proximal myopathy. CK(U/L): 510. MB: Vacuolar myopathy. MB: NI. ENMG: PSW, ﬁbrillation, CRD, myotonic discharges, myopathic MUPs	30% (idem previous). Low DBS Ez activity.	IVS1-13T>G (c32-13T>G); E888X 2662G>T	Compound heterozygous	1bi; 18														
Herbert et al., 2018 [87]	LOPD	Ninety patients. Cardiac involvement in Pompe disease associated with the c.32-13T>G variant was assessed by (1) medical record review. Clinical history, GAA variants, physical examination at clinical evaluations, cardiology evaluations including chest radiograph, electrocardiogram (ECG) and echocardiography (Echo)	NI	NI	83, all of them white, had the c.-32-13T>G variant present on at least 1 allele (92.22%).	Compound heterozygous	1bi	NI	U.S. resident	NI
					5 patients were c.-3213T>G; c.-3213T>G	Homozygous	1bi			
Torrealba-Acosta et al., 2017 [88]	LOPD	Patient who presented with progressive weakness, recurrent episodes of respiratory failure associated with pneumonia, a predominantly demyelinating mixed sensorimotor polyneuropathy, and paraspinal complex repetitive discharges. creatine kinase (CK) of 132 IU/L. Physical examination, neurological examination, motor examination. Babinski and Hofmann signs. Cardiologic examination: Electrocardiography. Needle electromyogram. Nerve conduction studies.	NI	DBS: reduced activity (neutral/acid: 73.0, reference: <30)	c.-32-13T>G; c.2560C>T; c.1551+42G>A	Compound heterozygous	1bi; e18; i10	NI	Costa Rican	NI
											Savarese et al., 2018 [68]	LOPD	Sixteen patients (from nine unrelated families) presented with limb-girdle phenotype (P1; P5b;c and P6b: fatigability; P2: myalgia; P3: fatigability, dysphagia; P5a,b,c,d: exercise intolerance; P5a; P6a and P8: fatigability myalgia; P7: cramps and P9: fatigability, cardiac involvement ) and/or an isolated hyperCKemia (P1; P2; P5a and b: 5-10X; P3; P4a;b;c;d; P5c; P6b;c; P7; and P8: 2.5-5X; P6a: normal; P9:NI). Clinically evaluated by neurologists. Functional measures (6-minute walking test, 6MWT), creatine kinase levels and respiratory functions (forced vital capacity, FVC, in supine and/or seated position). Electromyography. See Table 1.	NI	P1: lymphocytes (14>2) (pmol/min/mg)	c.784G>A; c.-32-13T>G	Compound heterozygous	4; 1bi	NI	Italian	NGS (MotorPlex) and Sanger sequencing
															P2: DBS:0 (pmol/min/mg)	c.1564C>G; c.-32-13T>G		11; 1bi			
P3: muscle: 11.7 (pmol/min/mg)	c.1927G>A; c.-32-13T>G	14; 1bi																			
P4a; b; c; d (all same family): NI	c.1124G>T; c.-32-13T>G	7; 1bi																			
P5a y b: DBS: 0.20; P5c: DBS: 0.08 (all same family) (pmol/min/mg)	c.1124G>T; c.-32-13T>G	7; 1bi																			
P6a: DBS: 0.24; P6b: DBS: 0.22; P6c: DBS: 0.16 (all same family) (pmol/min/mg)	c.2237G>A; c.-32-13T>G	16; 1bi																			
P7: DBS: 0.20 (pmol/min/mg)	c.784G>A; c.-32-13T>G	4; 1bi																			
P8: DBS: 0.13 (pmol/min/mg)	c.G989A; c.-32-13T>G	6; 1bi																			
P9: NI	c.1124G>T; c.-32-13T>G	7; 1bi																			
Liu et al., 2018 [38]	LOPD	P1: Initial symptoms: muscle weakness, CK (U/L): 1325.6 EMG: Myogenic damage. Muscle biopsy: Vacuolar myopathy	NI	NI	c.1201C>A; c.1057C>T	Compound heterozygous	8; 6	Unknown/unknown	Chinese	NGS (a gene panel that contains 142 neuromuscular disorder-related genes, including 15 genes responsible for glycogen storage disease) and Sanger sequencing
											P2: Initial symptoms: Muscle weakness of lower extremities, CK (U/L): 1168.3 EMG: Myogenic damage. Muscle biopsy: Vacuolar myopathy	c.2238G>C; c.2235dupG	16; 16	Potentially mild/unknown
		P3: Initial symptoms: Muscle weakness, CK (U/L): 73.5 EMG: Myogenic damage. Muscle biopsy: Vacuolar myopathy			c.1309C>T; c.2051C>A		8; 15	Less severe/unknown						
											P4: Initial symptoms: Muscle weakness of lower extremities, CK (U/L): 1652.0 EMG: Myogenic damage. Muscle biopsy: Vacuolar myopathy	c.2237G>A; c.796C>A	16; 4	Very severe/unknown
		P5: Initial symptoms: Muscle weakness, CK (U/L): 1652.0 EMG: Myogenic damage. Muscle biopsy: Vacuolar myopathy			c.1799G>C; c.1780C>T		13; 13	Unknown/unknown						
											Niño et al., 2019 [66]	Classified patients based on the criteria stated in [75]. Classic infantile PD: if they presented symptoms at or under 12 months of age. Childhood PD: The age of symptom onset was before 18 years of age was absent. Adult PD first symptoms presented at the age of 18 years or later.	Classic infantile PD: had evident signs of hypertrophic cardiomyopathy (cardiac enlargement visible on chest X‐ray, evidence of hypertrophy by echography).	Predicted CRIM status based on the type of variant for all variants. Variants that caused a frameshift were predicted to be CRIM negative. Missense variants were predicted to be CRIM positive, provided that no effect on splicing was predicted. Known and predicted splice variants were classified with an unknown CRIM status	NI	562 described variants, 422 of which are listed as disease‐associated.	NA	Along the gene. For introns, disease‐associated variants were found in all introns except for intron 5, without a clear pattern. See Table 1 for most common variants.	See the database www.pompevariantdatabase.nl	Caucasian; Asian; African/African American; Latin-American; Undetermined	NI
		Childhood PD: evident hypertrophic cardiomyopathy was absent. Adult PD: not clinical spectrum specified.																			
		Reuser et al., 2019 [40]			Classic		Patients were classified as Group A: Symptom onset ≤ 12 months of age with cardiomyopathy (typically classified as classic infantile Pompe disease);	Positive, negative, and unknown			NI	A total of 2,075 (1,205 exonic/870 intronic) GAA variants were reported among the 1,079 patients (Table 2). Of these, 392 variants were unique (as defined in Methods), 80 being novel (Table 3), and 312 variants (Table S1) being known.	NA	1205 exonic/870 intronic	See Table 3 for novel variants identified	Europe North America Asia‐Pacific Latin America Middle East	NI
					Childhood and Adult		Patients were classified as Group B: Symptom onset ≤ 12 years of age (includes patients with symptom onset ≤ 12 months of age without cardiomyopathy in the first year of life and not included in Group A); and r Group C: Symptom onset > 12 years of age. Pompe disease diagnosis reported as confirmed by enrolling physicians and with ≥ 1 documented pathogenic variant. Patients were excluded if dates of diagnosis and/or symptom onset were missing.	B: positive, negative, unknown; C: positive, unknown								B: Europe, North America, Asia‐Pacific, Latin America, Middle East; C: All except Middle East	
		Johnson et al., 2017 [39]			LOPD?		P3: Unexplained limb-girdle muscle weakness. Displayed a slowly progressive proximal lower limb and axial weakness. No respiratory insufficiency, and an EMG showed no abnormalities. Despite normal cardiac function, the patient was previously diagnosed with Brugada syndrome, a genetic cause for abnormal electrocardiogram findings that are associated with an increased risk of sudden cardiac death. Serum creatine kinase activity was measured at 1729 U/L (N < 190 U/L).	NI			DBS test: reduce activity	c.2331 +2T>A; c.-32-13T>G	Compound heterozygous	16; 1bi	NI	German	NGS: whole exome sequencing + Sanger sequence confirmation
P5: Unexplained limb-girdle muscle weakness. Back pain and elevated serum creatine kinase levels (1500 U/L), and retrospectively recognized mild proximal upper limb weakness when raising a load. The disease course progressed over the following nine years, with asymmetrical proximal weakness becoming more obvious. No respiratory insufficiency.	Normal to slightly lower		*c.2066_2070dupAGCCG*; c.-32-13T>G	Compound heterozygous		15; 1bi	NI		Romanian								
P7: Unexplained limb-girdle muscle weakness. Elevated serum creatine kinase activity (increased less than 10×). No respiratory insufficiency.	Deficiency		c.2269C >T; c.-32-13T>G	compound heterozygous		16; 1bi	NI		Serbian								
P8: Unexplained limb-girdle muscle weakness. Normal serum creatine kinase activity. No respiratory insufficiency.	Deficiency		c.2051C>G; c.-32-13T>G	Compound heterozygous		15; 1bi	NI		Serbian								
P17: Unexplained limb-girdle muscle weakness. Progressive proximal muscle weakness, pain, and fatigability in his fourth decade of life, but with normal levels of serum creatine kinase activity. He also displayed weakness of the back muscles and had notable respiratory dysfunction: his forced vital capacity was reduced to 2.41 (47% predicted normal value) in the sitting and to 2.0 l (39% predicted normal value) in the lying position. An EMG detected mild myopathic changes, and the muscle biopsy showed increased variability in fiber diameter without any abnormal glycogen accumulation.	Two DBS tests: the first suggested a reduction in α-glucosidase activity, while the second detected normal levels of enzyme activity. A subsequent analysis of α-glucosidase in lymphocytes and fibroblasts revealed borderline and low normal activity, respectively.		c.1192delC; c.2716G>A	Compound heterozygous		7; 19	NI		Caucasian								
P18: Unexplained limb-girdle muscle weakness. Proximal muscle weakness and a serum creatine kinase activity of 599 U/L (N < 170 U/L). The patient also showed a myopathic electromyogram (EMG). A muscle biopsy was indicative of a myofibrillar myopathy, with numerous vacuolated, degenerated, and atrophic fibers confirmed by NADH-tetrazolium reductase staining. The periphery of the vacuoles had an abnormal immunoreactivity for desmin and p62. The fiber-type proportions and distributions appeared normal; however, an ultrastructural examination displayed Z-band streaming with an accumulation of granular material. Glycogen accumulation in the vacuoles was noted, but the overall appearance was not suggestive of a glycogenosis. Respiratory insufficiency.	DBS test within the normal to lower ranges		c.525delT; c.-32-13T>G	Compound heterozygous		2; 1bi	NI		White British								
P32: Unexplained limb-girdle muscle weakness. Serum creatine kinase activity was persistently elevated; when first investigated at age 15, it was 662 U/L (N < 190 U/L). No cardiac involvement nor exhibited extreme respiratory distress, although a 13% reduction between sitting and supine forced vital capacity was observed. The patient described only slight dyspnea following physical exertion but otherwise considered himself healthy. A muscle biopsy and an EMG were mildly myopathic.	DBS test displayed reduced enzymatic activity. Persistently elevated, first measure: 662 U/L (N < 190 U/L)		c.525delT; c.-32-13T>G	Compound heterozygous		2; 1bi	NI		Caucasian								
P35: Unexplained limb-girdle muscle weakness. Rapidly progressive proximal lower limb weakness, difficulty in ascending stairs, and an inability to stand unaided from a supine position. The patient now has scoliosis, fatigability, paraspinal muscle atrophy, and proximal upper limb atrophy and weakness. Paraclinical examinations revealed a myopathic EMG, a dystrophic biopsy, and mildly elevated serum creatine kinase levels (increased less than 10×).	NI		c.569G>A; c.2020C>G	Compound heterozygous		3; 14	NI		Caucasian								
Peruzzo et al., 2019 [5]	IOPD and LOPD	NA	NA	NA	Interesting update about all mutations in GAA gene	-	All genes	All categories	All	NI
Ebrahimi et al., 2017 [89]	LOPD?	P1: Respiratory distress and muscle weakness; P2: respiratory distress	NI	Significantly decrease (range: 0.13–0.19 nmol/spot*21 h)	Revealed ten alterations in the patients. According to Pompe Center, one of the alterations was novel, and nine of them were previously reported. Novel mutation: c. 1650del G (**)	-	12	NI	Iranian	Sanger sequencing

Supplementary Material 2 

**Table 9:** Overview of the 96 articles included in the scoping review organized per year *In this table, we provide the reference numbers of articles we cited throughout the full text of our article. **When the DOI was unavailable, we provided either the article's PMID or ISSN. DOI, digital object identifier; ISSN, international standard serial number; JOPD, juvenile-onset Pompe disease; NA, not applicable; PMID, PubMed unique identifier; yrs., years

No. order	Year	Article first author and reference number*	Article title and DOI**	Language	Subtype of Pompe disease (PD)	Country of the included patients	Study design	Inclusion criteria	No. of patients with PD	Sex distribution of PD patients (M/F)	Estimated age of patients with PD
1	2017	Tsai et al. [33]	Next generation deep sequencing corrects diagnostic pitfalls of traditional molecular approach in a patient with prenatal onset of Pompe disease. 10.1002/ajmg.a.38333	English	IOPD	United States	Case report	NA	1	1/0	At birth
2	2017	Supriya et al.	Effect of temperature on lysosomal enzyme activity during preparation and storage of dried blood spots. 10.1002/jcla.22220	English	IOPD and LOPD	India	Cross-sectional	NA	12	NI	Between 25 and 30 yrs.
3	2017	Bar-Yoseph et al.	Cardiopulmonary exercise test to quantify enzyme replacement response in paediatric Pompe disease. 10.1002/ppul.23830	English	Pediatric PD	Israel	Case-control	PD diagnosed by a deficiency of acid alpha-glucosidase enzyme activity in dry blood spots and/or lymphocytes, followed by the study of the disease-causing mutations using DNA analysis of the GAA gene, age ≥8 y/o, ERT ≥1 year, capable of cycling on a stationary bicycle.	5	02-Mar	10-19 years, 4 infantile-onset, and 1 diagnosed at 5 yrs.
4	2017	Burlina et al.	Newborn screening for lysosomal storage disorders by tandem mass spectrometry in Northeast Italy. 10.1007/s10545-017-0098-3	English	IOPD and LOPD	Italy	Cross-sectional	NA	4	02-Mar	Newborns
5	2017	Bekircan-Kurt et al. [64]	New mutations and genotype–phenotype correlation in late-onset Pompe patients. 10.1007/s13760-016-0738-7	English	LOPD	Turkey	Case series	NA	7	02-May	Range 17–37 yrs.
6	2017	Calabrese et al.	Síntomas atípicos compatibles con fibromialgia dilatan el diagnóstico en paciente con enfermedad de Pompe de inicio tardío. 10.1016/j.neuarg.2016.09.004	Spanish	LOPD	Argentina	Case report	NA	1	0/1	52 yrs.
7	2017	Schänzer et al.	Quantification of muscle pathology in infantile Pompe disease. 10.1016/j.nmd.2016.10.010	English	Classic infantile PD and early childhood	Germany	Cohort	Children with infantile PD confirmed by significantly reduced GAA activities in fibroblasts or lymphocytes, and by detection of GAA mutations in both alleles.	Classic Infantile (CI): 11 Early childhood (ECh): 2	CI: 3/8 Ech: 1/1	CI: 1.5 to 136 months Ech: 49/4 months
8	2017	Golsari et al.	Prevalence of adult Pompe disease in patients with proximal myopathic syndrome and undiagnosed muscle biopsy. 10.1016/j.nmd.2017.12.001	English	Adult PD	Germany	Cross-sectional	Existence of a limb-girdle myopathic weakness (LGMW) or isolated hyperCKemia and undiagnosed muscle biopsy.	2	01-Jan	29/22 yrs.
9	2017	Kim et al.	Severe trismus due to bilateral elongated mandibular coronoid process in an infantile-onset Pompe disease patient: a case report. 10.1016/j.pedex.2017.03.002	English	IOPD	Korea	Case report	NA	1	0/1	5-month-old infant
10	2017	Guimarães et al.	Prevalence of late-onset Pompe disease in Portuguese patients with diaphragmatic paralysis - DIPPER study. 10.1016/j.rppnen.2017.02.004	English	LOPD	Portugal	Cross-sectional	Patients with a diagnosis of diaphragmatic paralysis of unknown etiology.	4	NI	NI
11	2017	Chan et al.	Elevated creatinine kinase in a 6-year-old boy. 10.1016/j.spen.2017.03.003	English	JOPD	Australia	Case report	NA	1	1/0	6-yrs.
12	2017	Chan et al.	The emerging phenotype of late-onset Pompe disease: a systematic literature review. 10.1016/j.ymgme.2016.12.004	English	LOPD	Not Applicable	Systematic review	Articles that provided data on the characteristics of LOPD identified via the PubMed database published since the approval of ERT in 2006.	NA	NA	NA
13	2017	Navarrete-Martínez et al. [47]	Newborn screening for six lysosomal storage disorders in a cohort of Mexican patients: three-year findings from a screening program in a closed Mexican health system. 10.1016/j.ymgme.2017.03.001	English	IOPD and LOPD	Mexico	Cross-sectional	NA	11	NI	Newborns
14	2017	Rairikar et al. [83]	Insight into the phenotype of infants with Pompe disease identified by newborn screening with the common c.-32-13T>G late-onset GAA variant. 10.1016/j.ymgme.2017.09.008	English	LOPD	United States	Case series	NA	7	03-Apr	0.4-4.0 yrs.
15	2017	Chen et al. [84]	Clinical and molecular characterization of infantile-onset Pompe disease in Mainland Chinese patients: identification of two common mutations. 10.1089/gtmb.2016.0424	English	IOPD	China	Cross-sectional	All patients diagnosed with IOPD between 2013 and 2016 at Shanghai Children’s Medical Center were included.	25 unrelated	14-Nov	Median age 3.4 months (range: 1.0–7.1 months)
16	2017	Torrealba-Acosta et al. [88]	First clinical and genetic description of a family diagnosed with late-onset Pompe disease from Costa Rica. 10.1016/j.nmd.2017.06.010	English	LOPD	Costa Rica	Case report	NA	1	1/0	50 yrs.
17	2017	Eleftheriadis et al.	Late-onset Pompe’s disease in a haemodialysis patient: a first case report. 10.1111/hdi.12617	English	LOPD	Greece	Case report	NA	1	1/0	37 yrs.
18	2017	Ripolone et al.	Effects of short-to-long term enzyme replacement therapy (ERT) on skeletal muscle tissue in late onset Pompe disease (LOPD). 10.1111/nan.12414	English	LOPD	Italy	Case series	NA	18	08-Oct	Mean: 36.8 yrs. (SD: 13.2)
19	2017	Ceyhan et al.	An 18-Month-Old Child with Infantile Pompe Disease: Oral Signs. 10.1155/2017/5685941	English	IOPD	Turkey	Case report	NA	1	1/0	18 months
20	2017	Eyskens et al.	Newborn screening for lysosomal storage disorders in Belgium. The importance of sex-and age-dependent reference ranges. 10.1177/2326409817744231	English	IOPD and LOPD	Belgium	Cross-sectional	DBS samples from 20,000 newborns collected in the period from June 2015 to February 2016.	NI	NI	NI
21	2017	Johnson et al. [39]	Identification of GAA variants through whole exome sequencing targeted a cohort of 606 patients with unexplained limb-girdle muscle weakness. 10.1186/s13023-017-0722-1	English	IOPD and LOPD	United Kingdom	Cross-sectional	Unexplained limb-girdle muscle weakness and/or elevated serum creatine kinase activity.	8	05-Mar	NI
22	2017	Kuperus et al.	Long-term benefit of enzyme replacement therapy in Pompe disease. 10.1212/wnl.0000000000004711	English	LOPD	Netherlands	Cohort	NI	102	NI	NI
23	2017	Liao et al.	Mass spectrometry but not fluorometry distinguishes affected and pseudodeficiency patients in newborn screening for Pompe disease. 10.1373/clinchem.2016.269027	English	IOPD and LOPD	Taiwan	Cross-sectional	Newborn DBS were collected and shipped to Taiwan within three days of birth at ambient temperature.	IOPD = 11; LOPD = 12	NI	Newborns
24	2017	Kronn et al. [24]	Management of confirmed newborn screened patients with Pompe disease across the disease spectrum. 10.1542/peds.2016-0280E	English	LOPD	NA	Guidelines	NA	NA	NA	NA
25	2017	Sixel et al.	Respiratory manifestations in late-onset Pompe disease: a case series conducted in Brazil. 10.1590/s1806-37562015000000343	English	LOPD	Brazil	Cross-sectional	Patients with a definitive molecular diagnosis of LOPD.	6 (two related)	04Feb	Median age was 15 yrs. (range, 13-50 yrs.)
26	2017	Ebrahimi et al. [89]	Identification of a novel mono nucleotide deletion mutation in GAA in Pompe disease patients. 10.4103/jrms.JRMS_874_16	English	LOPD	Iran	Cross-sectional	NA	2 (related)	1/1	26/34 yrs.
27	2017	Kumar et al.	Unusual presentation of atypical infantile Pompe disease in the newborn period with left ventricular hypertrophy. 10.7860/jcdr/2017/20756.9849	English	IOPD	India	Case report	NA	1	0/1	Newborn
28	2017	Walczak-Galezewska et al.	Late-onset Pompe disease in a 54-year-old sports man with an episode of syncope: a case report. PMID 28925476	English	LOPD	Poland	Case report	NA	1	0/1	54 yrs.
29	2018	Li et al. [82]	Early prenatal diagnosis of lysosomal storage disorders by enzymatic and molecular analysis. 10.1002/pd.5329	English	IOPD	China	Cross-sectional	Pregnant women receiving prenatal diagnosis of LSDs from February 2013-May 2017 in Guangzhou Women and Children's Medical Centre	7	NI	Prenatal
30	2018	Scheidegger et al.	36-Months follow-up assessment after cessation and resuming of enzyme replacement therapy in late onset Pompe disease: data from the Swiss Pompe Registry. 10.1007/s00415-018-9065-7	English	LOPD	Switzerland	Cohort	Patients with genetically confirmed LOPD.	7	03-Apr	NI
31	2018	van der Meijden et al.	Long-term follow-up of 17 patients with childhood Pompe disease treated with enzyme replacement therapy. 10.1007/s10545-018-0166-3	English	Classic infantile PD	Multicentre Study (Netherlands, Belgium, Germany, UK, USA)	Cross-sectional	Children with a confirmed diagnosis of Pompe disease in whom ERT had been initiated before the age of 18 years.	17	11-Jun	Between 1.1 and 16.4 yrs. (median 11.9 yrs.)
32	2018	Moravej et al. [85]	A new mutation causing severe infantile onset Pompe disease responsive to enzyme replacement therapy. PMID 29749992	English	IOPD	Iran	Case report	NA	1	0/1	Neonate
33	2018	Semplicini et al. [86]	Late-onset Pompe disease in France: molecular features and epidemiology from a nationwide study. 10.1007/s10545-018-0243-7	English	LOPD	France	Cross-sectional	(1) Confirmed diagnosis of PD, established with at least two different methods (either enzymatic test on lympho/leukocytes and confirmation on fibroblasts, or enzymatic test on lympho/leukocytes and confirmation by GAA gene sequencing, or enzymatic test on dried-blood spots and confirmation by GAA gene sequencing); (2) disease onset ≥ 2 years of age.	246	116/130	Mean age of 43 yrs.
34	2018	Herbert et al. [87]	Severe cardiac involvement is rare in patients with late-onset Pompe disease and the common c.-32-13T>G variant: implications for newborn screening. 10.1016/j.jpeds.2018.02.007	English	LOPD	United States	Cross-sectional/narrative review	(1) Medical record review of the Duke Pompe disease patient cohort, (2) literature review, and (3) review of NBS data from programs in the states of Missouri, Illinois, and New York.	83	33/50	48 yrs. (range 0.5-78 yrs.)
35	2018	Hossain et al.	A case of adult-onset Pompe disease with cerebral stroke and left ventricular hypertrophy. 10.1016/j.jstrokecerebrovasdis.2018.06.043	English	LOPD	Japan	Case report	NA	1	0/1	59 yrs.
36	2018	Savarese et al. [68]	Targeted gene panel screening is an effective tool to identify undiagnosed late onset Pompe disease. 10.1016/j.nmd.2018.03.011	English	LOPD	Italy	Cross-sectional	Patients with a broad range of myopathies, including LGMDs, congenital myopathies, distal myopathies, and isolated hyperCKemia, were recruited in the network of the Italian Association of Myology	17	10-Jun	Range 33-71 yrs.
37	2018	Guevara-Campos et al. [65]	Skeletal alterations, developmental delay and new mutations in juvenile-onset Pompe’s disease. 10.1016/j.nmd.2018.11.013	English	JOPD	Venezuela	Case series	NA	3	3/0	Age 6-14 yrs.
38	2018	McIntosh et al.	Neuroimaging findings in infantile Pompe patients treated with enzyme replacement therapy. 10.1016/j.ymgme.2017.10.005	English	IOPD	United States	Cross-sectional	Patients evaluated between 1999 and 2015 who had undergone brain imaging with CT or MRI as part of clinical care. All patients had a confirmed diagnosis of Pompe disease.	23	16-Jul	Age 2-38 months
39	2018	Herbert et al.	Early-onset of symptoms and clinical course of Pompe disease associated with the c.-32–13T>G variant. 10.1016/j.ymgme.2018.08.009	English	LOPD	United States	Case series	Medical records of 84 LOPD patients followed at Duke University Medical Center (DUMC) with the c.-32-13T>G variant were reviewed to identify patients who were clinically diagnosed with Pompe disease. Patients with symptom onset in the first two years of life and receiving ERT were included.	4	02-Feb	Ranged from 10 months to 26 months
40	2018	Fukuhara et al.	A molecular analysis of the GAA gene and clinical spectrum in 38 patients with Pompe disease in Japan. 10.1016/j.ymgmr.2017.10.009	English	IOPD and LOPD	Japan	Case series	Patients with Pompe disease were analyzed from 2008 to 2016.	38	22/15/NI (1)	Median age of onset of IOPD: 4 months (range: 0–12 months). Median age of onset of LOPD patients: 11 yrs. (range: 1–72 yrs.)
41	2018	Pappa et al.	Renal artery fibromuscular dysplasia in Pompe disease: a case report. 10.1016/j.ymgmr.2018.07.002	English	LOPD	France	Case report	NA	1	1/0	42 yrs.
42	2018	Tortorelli et al. [30]	Moonlighting newborn screening markers: the incidental discovery of a second-tier test for Pompe disease. 10.1038/gim.2017.190	English	IOPD	United States	Cross-sectional	NA	NA	NA	Newborns
43	2018	Figueroa-Bonaparte et al. [43]	Quantitative muscle MRI to follow up late onset Pompe patients: a prospective study. 10.1038/s41598-018-29170-7	English	LOPD	Spain	Cohort	NI	32	14/18	Ranged 8-65 yrs.
44	2018	Kakkar et al.	Pompe disease: an Indian series diagnosed on muscle biopsy by ultrastructural characterization. 10.1080/01913123.2018.1447624	English	IOPD and LOPD	India	Case series	All cases diagnosed as PD on muscle biopsy were retrieved from the database of the Pathology Laboratory at the All-India Institute of Medical Sciences and reviewed, retrospectively.	19	12Jul	Mean age of the entire patient cohort was 10.4 yrs.
45	2018	Favejee et al.	Association of muscle strength and walking performance in adult patients with Pompe disease. 10.1093/ptj/pzy090	English	LOPD	Netherlands	Cross-sectional	Adult patients with a confirmed diagnosis of Pompe disease were included in this study. All patients were first seen between December 2003 and August 2012 at the Center for Lysosomal and Metabolic Diseases of the Erasmus MC University Medical Center, Rotterdam.	108	55/53	Median age of 50 yrs. (minimum = 25 y; maximum = 76 y) and had symptoms for a median of 15 yrs.
46	2018	Ebbink et al. [70]	Classic infantile Pompe patients approaching adulthood: a cohort study on consequences for the brain. 10.1111/dmcn.13740	English	Classic infantile PD	Netherlands	Cohort	Oldest classic infantile patients of our current cohort (start of ERT between 1999 and 2009).	11	NI	Aged from 7 yrs. 7 months to 17 yrs. 8 month
47	2018	Musumeci et al.	Central nervous system involvement in late-onset Pompe disease: clues from neuroimaging and neuropsychological analysis. 10.1111/ene.13835	English	LOPD	Italy	Cross-sectional	Patients with defined diagnosis of LOPD, divided into two groups: one with pre-symptomatic hyperCKemia with no muscle weakness and the second with limb-girdle muscle weakness.	21	10-Nov	mean age 49 ± 18.4 yrs.
48	2018	Owens et al.	Infantile-onset Pompe disease: a case series highlighting early clinical features, spectrum of disease severity and treatment response. 10.1111/jpc.14070	English	IOPD	Australia	Case series	NA	4	01-Mar	Age 3–9 months
49	2018	Elenga et al.	Incidence of infantile Pompe disease in the Maroon population of French Guiana. 10.1136/bmjpo-2017-000182	English	IOPD	French Guiana	Cross-sectional	All patients with IPD diagnosed on an enzymatic basis, from January 2002 to December 2015 (13 years).	19	NI	Newborns
50	2018	Hensel et al.	Decreased outlet angle of the superior cerebellar artery as indicator for dolichoectasia in late onset Pompe disease. 10.1186/s13023-018-0794-6	English	LOPD	Germany	Case-control	Twenty LOPD patients with genetically and biochemically confirmed diagnoses of Pompe disease	20	10-Oct	Age 53.7 ± 14.6 yrs., range 19 to 81 yrs.
51	2018	Menzella et al.	Acute respiratory failure as presentation of late-onset Pompe disease complicating the diagnostic process as a labyrinth: a case report. 10.1186/s40248-018-0145-4	English	LOPD	Italy	Case report	NA	1	0/1	52 yrs.
52	2018	Lollert et al.	Quantification of intramuscular fat in patients with late-onset Pompe disease by conventional magnetic resonance imaging for the long-term follow-up of enzyme replacement therapy. 10.1371/journal.pone.0190784	English	LOPD	Germany	Cohort	Patients diagnosed with late-onset Pompe disease from 2006 through 2015, presenting at the metabolic diseases' clinic (Villa Metabolica) of the Medical Center of the Johannes Gutenberg University, Mainz, Germany.	41	22/19	Median age: 28 yrs., (range: 6–74 yrs.)
53	2018	Lorenzoni et al.	Late-onset Pompe disease: what is the prevalence of limb-girdle muscular weakness presentation? 10.1590/0004-282x20180018	English	LOPD	Brazil	Cross-sectional	Patients with unexplained limb-girdle muscular weakness without vacuolar myopathy in their muscle biopsy, no affected relatives, and no definitive diagnosis of limb-girdle muscular weakness at the Hospital de Clínicas of the Federal University of Paraná (Curitiba, Brazil).	24	15-Sep	Varied between one and 57 yrs., with a mean of 21.8 ± 17.8 yrs.
54	2018	Remiche et al.	Low prevalence estimates of late-onset glycogen storage disease type II in French-speaking Belgium are not due to missed diagnoses. 10.3233/JND-180336	English	LOPD	Belgium	Cohort	Adult patients (>18 years old) with an 80 unexplained muscle phenotype. The phenotype categories were stratified as weakness, muscle pain, 82 muscle fatigability, asymptomatic high CK level, 83 acute rhabdomyolysis, and muscle atrophy.	2	NI	NI
55	2018	Nascimento et al.	Nuevo fenotipo de enfermedad de Pompe infantil. 10.33588/rn.6604.2017492	Spanish	IOPD	Spain	Case report	NA	2	01-Jan	Breastfeeding infants: 2 and 3 months
56	2018	Chiang et al.	Performance of the four-plex tandem mass spectrometry lysosomal storage disease newborn screening test: the necessity of adding a 2nd tier test for Pompe disease. 10.3390/ijns4040041	English	IOPD and LOPD	Taiwan	Cross-sectional	NA	6 (IOPD:1/LOPD:5)	NI	Newborns
57	2018	Liu et al. [38]	Identification of seven novel mutations in the acid alpha-glucosidase gene in five Chinese patients with late-onset Pompe disease. 10.4103/0366-6999.225056	English	LOPD	China	Case series	Clinical and pathological data of patients diagnosed with glycogen storage disease at our institution from April 1986 to August 2017 were collected at the Army General Hospital, China.	5	03-Feb	Ranged from 1 to 19 yrs. with a median of 13 yrs.
58	2018	Alvarez Bertea et al.	Ecografía diafragmática y pruebas de función pulmonar en la enfermedad de Pompe. Presentación de casos clínicos. ISSN1852-236X	Spanish	LOPD	Argentina	Case series	NA	4 (3 related)	03-Jan	Ranged from 39-76 yrs.
59	2019	Lee et al.	Ultrasonography-based qualitative and quantitative evaluation approaches for Pompe disease. 10.1007/s40846-019-00502-w	English	IOPD and LOPD	Taiwan	Case-control	NI	23	NI	Aged 1 to 19 yrs.
60	2019	Niño et al. [66]	Extension of the Pompe mutation database by linking disease‐associated variants to clinical severity. 10.1002/humu.23854	English	IOPD and LOPD	Not Applicable	Case series	NA	867	NI	NA
61	2019	Reuser et al. [40]	GAA variants and phenotypes among 1,079 patients with Pompe disease: Data from the Pompe Registry. 10.1002/humu.23878	English	IOPD and LOPD	Multicentre Study (Europe, North America, Asia‐Pacific, Latin America, and the Middle East)	Case series	NA	1079	NI	NA
62	2019	Berger et al.	Forced vital capacity and cross‑domain late‑onset Pompe disease outcomes: an individual patient‑level data meta‑analysis. 10.1007/s00415-019-09401-1	English	LOPD	Multicentre Study (Danish, French, Swiss, Greek, Polish, German and Italy)	Systematic review	Eligible studies were those reporting individual patient-level data on LOPD patients, regardless of treatments, with FVC and at least one other LOPD outcome.	276	46.5% of males in trials reporting sex	Mean age was 42.2 yrs. (SD: 16.3 yrs.)
63	2019	Bergsma et al. [36]	A genetic modifier of symptom onset in Pompe disease. 10.1016/j.ebiom.2019.03.048	English	IIOPD and LOPD	Multicentre Study (Netherlands, Germany, Italy)	Cross-sectional	Patients enrolled in this study were all diagnosed with Pompe disease on the basis of GAA enzyme deficiency and the presence of two disease-associated variants in the GAA gene, at least one of which was c.-32-13TNG.	143	NII	NI
64	2019	Spiesshoefer et al.	The nature of respiratory muscle weakness in patients with late-onset Pompe disease. 10.1016/j.nmd.2019.06.011	English	LOPD	Germany	Case-control	Only adult patients with genetically proven LOPD and ongoing ERT were recruited.	13	07-Jun	Age 51±6 yrs.
65	2019	Tsai et al.	Clinical features of Pompe disease with motor neuronopathy. 10.1016/j.nmd.2019.09.011	English	IOPD	Taiwan	Case series	Patients with Pompe disease who received long-term enzyme replacement therapy.	7	06-Jan	Age was 9.3 ± 1.8 yrs. (7-11 yrs.)
66	2019	Kishnani et al. [35]	Clinical characteristics and genotypes in the ADVANCE baseline dataset, a comprehensive cohort of US children and adolescents with Pompe disease. 10.1038/s41436-019-0527-9	English	IOPD and LOPD	United States	Clinical trial	Aged ≥1 year with confirmed Pompe disease previously receiving 160 L glucosidase alfa were eligible.	113	60/53	Aged 1-18 yrs.
67	2019	Fernández-Simón et al. [37]	PDGF-BB serum levels are decreased in adult onset Pompe patients. 10.1038/s41598-018-38025-0	English	LOPD	Spain	Case-control	Thirty-seven AOPD patients were included in the study	37 (29: Symptomatic; 8 Asymptomatic)	19/18	Symptomatic: 51 (31–65) yrs.; Asymptomatic 21 (8–51) yrs.
68	2019	Smith et al.	Dynamic respiratory muscle function in late-onset Pompe disease. 10.1038/s41598-019-54314-8	English	LOPD	United States	Case-control	NI	7	02-May	Age was 43.6 (± 12.8 yrs.)
69	2019	Hamed et al.	Mobility assessment using wearable technology in patients with late-onset Pompe disease. 10.1038/s41746-019-0143-8	English	LOPD	United States	Case series	LOPD patients self-reporting LOPD, ≥18 years, US residents, walking (with or without aid), and not on invasive ventilation, were recruited for a 6- to 8-week wearable study via patient organizations.	29	26-Mar	Mean age was 43 yrs.
70	2019	Jastrzębska et al.	Screening for late-onset Pompe disease in Poland. 10.1111/ane.13133	English	LOPD	Poland	Cross-sectional	Patients with limb-girdle muscle weakness, persistently elevated CK, rigid spine syndrome, dyspnea, myalgia, or siblings of the patient diagnosed with LOPD were included.	10	04-Jun	Mean age 32.3 yrs. (ranged 2-80 yrs., median 31)
71	2019	Meznaric et al.	Selective screening of late-onset Pompe disease (LOPD) in patients with non-diagnostic muscle biopsies. 10.1136/jclinpath-2018-205446	English	LOPD	Slovenia	Cross-sectional	Six hundred biopsies were recorded at the Neuromuscular Tissue Bank of the University of Ljubljana for the period 2004-2014.	0	0	NA
72	2019	Reynolds et al.	Identification of patients with Pompe disease using routine pathology results: PATHFINDER (creatine kinase) study. 10.1136/jclinpath-2019-205711	English	IOPD and LOPD	United Kingdom	Cross-sectional	Patients with elevated CK results (>2× upper limit of normal) identified in six hospital pathology databases.	1	NI	NI
73	2019	Ngiwsara et al.	Clinical course, mutations and its functional characteristics of infantile-onset Pompe disease in Thailand. 10.1186/s12881-019-0878-8	English	IOPD	Thailand	Cross-sectional	Patients with IOPD based on clinical and/or enzymatic diagnosis during 2000-2018 in the participating hospitals were enrolled.	12	09-Mar	Average was 4.1 months (range 2–8 months)
74	2019	Confalonieri et al. [18]	Is early detection of late-onset Pompe disease a pneumologist’s affair? A lesson from an Italian screening study. 10.1186/s13023-019-1037-1	English	LOPD	Italy	Cross-sectional	Patients with suspected LOPD according to a predefined clinical algorithm. From February 2015 to December 2017, 140 patients (57 ± 16 yrs., 80 males) were recruited in 19 Italian pneumological units.	2	NI	NI
75	2019	Zhao et al.	Characteristics of Pompe disease in China: a report from the Pompe registry. 10.1186/s13023-019-1054-0	English	IOPD and LOPD	China	Narrative review	Chinese patients enrolled in the Registry between Jan 2013 and 2 Sep 2016 with late-onset Pompe disease (LOPD; presentation after 12 months of age or presentation at ≤12 months without cardiomyopathy) were included.	59	NI	Age was 14.9 (12.35) yrs.
76	2019	Harlaar et al.	Large variation in effects during 10 years of enzyme therapy in adults with Pompe disease. 10.1212/wnl.0000000000008441	English	LOPD	Netherlands and France	Cohort	Thirty patients from the Netherlands and France who had started ERT during the only randomized placebo-controlled clinical trial with ERT in late-onset Pompe disease (NCT00158600) or its extension (NCT00455195) in 2005 to 2008.	30	14/16	49(41-60) yrs.
77	2019	Torres-Cuenca et al.	Functional assessment using short test in a patient with Pompe disease receiving enzyme replacement therapy: case report. 10.15446/cr.v5n2.76711	English	LOPD	Colombia	Case report	NA	1	0/1	34 yrs.
78	2019	Peruzzo et al. [5]	Molecular genetics of Pompe disease: a comprehensive overview. 10.21037/atm.2019.04.13	English	IOPD and LOPD	Not applicable	Narrative review	Review the spectrum of GAA mutations, their distribution in different populations, and their classification according to their impact on the GAA splicing process, protein expression, and activity.	NA	NA	NA
79	2019	Hahn et al.	Long-term out come and unmet needs in infantile-onset Pompe disease. 10.21037/atm.2019.04.70	English	IOPD	Not applicable	Narrative review	NI	NA	NA	NA
80	2019	Kim et al.	Clinical and molecular characterization of Korean children with infantile and late-onset Pompe disease: 10 years of experience with enzyme replacement therapy at a single center. 10.3345/kjp.2018.06968	English	IOPD and LOPD	Korea	Case series	Patients previously diagnosed by measurement of acid alpha-glucosidase activity in peripheral blood leukocytes or skeletal muscle biopsy specimens and by mutation analysis.	5	02-Mar	Patient 1: 2 months; Patients 2 and 3: 9 months; Patients 4: 17 yrs. Five months and Patient 5: 2 yrs. 9 months.
81	2019	Faverio et al.	Molecular pathways and respiratory involvement in lysosomal storage diseases. 10.3390/ijms20020327	English	IOPD and LOPD/LSD	Not applicable	Narrative review	Studies from 1968 through November 2018 investigated the respiratory manifestations and molecular pathways affected by LSD.	NA	NA	NA
82	2019	Burlina et al.	Implementation of second-tier tests in newborn screening for lysosomal disorders in North Eastern Italy. 10.3390/ijns5020024	English	IOPD and LOPD/LSD	Italy	Cross-sectional	Recalled 138 neonates (0.12%) for collection of a second dried blood spot.	8	06-Feb	Neonates
83	2019	Janeczko et al.	Current pharmacotherapy and diagnostic methods of Pompe Disease in Poland. 10.5281/zenodo.3457463	English	IOPD and LOPD	Not applicable	Narrative review	Most current research and information about Pompe disease can be found on Google Scholar and on the Science Direct website.	NA	NA	NA
84	2020	Yamauchi et al.	Potential patient screening for late-onset Pompe disease in suspected sleep apnea: a rationale and study design for a Prospective Multicenter Observational Cohort Study in Japan (PSSAP-J Study). 10.1007/s11325-020-02170-6	English	LOPD	Japan	Cohort	Patients who visit sleep labs at the participating institutions by themselves and who are referred by general practitioners and/or other specialists, including neurologists, for the purpose of the evaluation of any sleep problems.	NA	NA	NA
85	2020	Hussain et al.	Other Myopathies. 10.1016/j.ncl.2020.04.002	English	LOPD	USA	Case report	NA	1	0/1	48-yrs.
86	2020	Turco et al.	Nystagmus in Infantile Pompe Disease: a new feature? 10.23750/abm.v91i3.8366	English	IOPD	Italy	Case report	NA	1	0/1	3 month
87	2020	Gupta et al.	A race against time-changing the natural history of CRIM negative infantile Pompe disease. 10.3389/fimmu.2020.01929	English	IOPD	USA	Case report	NA	1	0/1	4 yrs.
88	2020	Paoletti et al.	Multicentric retrospective evaluation of five classic infantile Pompe disease subjects under enzyme replacement therapy with early infratentorial involvement. 10.3389/fneur.2020.569153	English	IOPD	Italy	Case series	Subjects had the classic form of Pompe disease and were CRIM-positive.	5	NI	One month to 16.5 yrs.
89	2020	Ullah et al. [34]	Identification of two novel variants in GAA under lying infantile-onset Pompe disease in two Pakistani families. 10.1515/jpem-2019-0477	English	IOPD	Pakistan	Case series	NA	2	2/0	3 month
90	2021	van den Dorpel et al.	Is the brain involved in patients with late-onset Pompe disease? 10.1002/jimd.12469	English	LOPD	Netherlands	Case series	Children and adults with a confirmed diagnosis of late-onset Pompe disease.	19	NI	11 to 56 yrs.
91	2021	Wenninger et al.	The impact of interrupting enzyme replacement therapy in late-onset Pompe disease. 10.1007/s00415-021-10475-z	English	LOPD	Germany	Cohort	Patients with late-onset Pompe disease who discontinued ERT due to the COVID-19 pandemic in Munich, Germany.	12	05-Jul	NI
92	2021	Starbuck et al.	Are there common walking gait characteristics in patients diagnosed with late-onset Pompe disease? 10.1016/j.humov.2021.102777	English	LOPD	United Kingdom	Case-control	Patients with LOPD were recruited from the Metabolic Unit at the Salford Royal NHS Foundation Trust. They were recruited for the study if they met the following criteria: 1) aged between 16 and 70 years, 2) had a positive diagnosis of LOPD, and 3) were able to walk 50 m or more continuously without the use of a walking aid	12	06-Jun	44.42 ± 13.96 yrs.
93	2021	Wasserstein et al. [53]	The future of newborn screening for lysosomal disorders. 10.1016/j.neulet.2021.136080	English	IOPD and LPD/LSD	USA	Narrative review	NA	NA	NA	NA
94	2021	Byrne et al.	Cardiac responses in paediatric Pompe disease in the ADVANCE patient cohort. 10.1017/S1047951121002079	English	Pediatric PD	USA	Clinical trial	ADVANCE methodology	NA	NA	NA
95	2021	Schlaeger et al.	Quantitative muscle MRI in patients with neuromuscular diseases—association of muscle proton density fat fraction with semi-quantitative grading of fatty infiltration and muscle strength at the thigh region. 10.3390/diagnostics11061056	English	LOPD	Germany	Cohort	NI	3	02-Jan	69.8 ± 19.2 yrs.
96	2021	Korlimarla et al.	New insights into gastrointestinal involvement in late-onset Pompe disease: lessons learned from bench and bedside. 10.3390/jcm10153395	English	LOPD	USA	Cohort	Adults (ages ≥ 18 years) with a confirmed diagnosis of LOPD who were evaluated at Duke between April 2017 and July 2018	58	23/35	Median age = 51.5 ± 15.5 yrs. (age range: 18-79 yrs.)

Supplementary Material 3

**Table 10:** Search strategy per database

Date	Database	Search strategy	No. of citations retrieved
Feb 8, 2022	PubMed/Medline (National Library of Medicine, NCBI)	Search	714
		Pompe [Title/Abstract] OR Acid Alpha-Glucosidase Deficiency [Title/Abstract] OR Acid Maltase Deficiency [Title/Abstract] OR GAA Deficiency [Title/Abstract] OR Glycogen Storage Disease Type II[Title/Abstract] OR GSD II [Title/Abstract] OR Glycogenosis Type II [Title/Abstract]	
Feb 9, 2022	ScienceDirect (Elsevier)	Search	1059
		Title, Abstract, Keywords	
		Pompe OR Acid Alpha-Glucosidase Deficiency OR Acid Maltase Deficiency OR GAA Deficiency OR Glycogen Storage Disease Type II OR GSD II OR Glycogenosis Type II	
		2017-2022	
Feb 9, 2022	Ingenta Connect (Ingenta)	Search	0
		Title, Abstract, Keywords	
		Pompe OR Acid Alpha-Glucosidase Deficiency OR Acid Maltase Deficiency OR GAA Deficiency OR Glycogen Storage Disease Type II OR GSD II OR Glycogenosis Type II	
		2017-2022	
Feb 9, 2022	Virtual Health Library (Latin American and Caribbean Center on Health Sciences Information, World Health Organization)	Search	23
		Title, Abstract, Subject	
		Pompe OR Acid Alpha-Glucosidase Deficiency OR Acid Maltase Deficiency OR GAA Deficiency OR Glycogen Storage Disease Type II OR GSD II OR Glycogenosis Type II	
		2017-2022	
Feb 9, 2022	Springer Link (Springer Nature)	Search	61
		Pompe OR Acid Alpha-Glucosidase Deficiency OR Acid Maltase Deficiency OR GAA Deficiency OR Glycogen Storage Disease Type II OR GSD II OR Glycogenosis Type II	
		2017-2022	
Feb 9, 2022	Wiley Online Library (John Wiley & Sons, Inc.)	Search	462
		Any field	
		Pompe OR Acid Alpha-Glucosidase Deficiency OR Acid Maltase Deficiency OR GAA Deficiency OR Glycogen Storage Disease Type II OR GSD II OR Glycogenosis Type II	
		2017-2022	
		Medical Science	
Feb 9, 2022	SAGE Journals (SAGE Publications)	Search	92
		Any field	
		Pompe OR Acid Alpha-Glucosidase Deficiency OR Acid Maltase Deficiency OR GAA Deficiency OR Glycogen Storage Disease Type II OR GSD II OR Glycogenosis Type II	
		2017-2022	
Feb 9, 2022	Scielo (Web of Science, Clarivate)	Search	14
		Title and Abstract	
		Pompe OR Acid Alpha-Glucosidase Deficiency OR Acid Maltase Deficiency OR GAA Deficiency OR Glycogen Storage Disease Type II OR GSD II OR Glycogenosis Type II	
		2017-2022	
Feb 10, 2022	Epistemonikos (Epistemonikos Foundation)	Search	40
		Title and Abstract	
		Pompe OR Acid Alpha-Glucosidase Deficiency OR Acid Maltase Deficiency OR GAA Deficiency OR Glycogen Storage Disease Type II OR GSD II OR Glycogenosis Type II	
		2017-2022	
Feb 10, 2022	Cochrane Library (John Wiley & Sons, Inc)	Search	0
		Title and Abstract	
		Pompe OR Acid Alpha Glucosidase Deficiency OR Acid Maltase Deficiency OR GAA Deficiency OR Glycogen Storage Disease Type II OR GSD II OR Glycogenosis Type II	
Feb 10, 2022	Directory of Open Access Journals (DOAJ)	Search	28
		Abstract	
		Pompe OR Acid Alpha Glucosidase Deficiency OR Acid Maltase Deficiency OR GAA Deficiency OR Glycogen Storage Disease Type II OR GSD II OR Glycogenosis Type II	

## References

[1] Marques JS (2022). The clinical management of Pompe disease: a pediatric perspective. Children (Basel).

[2] Taverna S, Cammarata G, Colomba P (2020). Pompe disease: pathogenesis, molecular genetics and diagnosis. Aging (Albany NY).

[3] (2023). Variant database. https://www.pompevariantdatabase.nl/pompe_mutations_list.php.

[4] de Faria DO, 't Groen SL, Hoogeveen-Westerveld M, Nino MY, van der Ploeg AT, Bergsma AJ, Pijnappel WW (2021). Update of the Pompe variant database for the prediction of clinical phenotypes: novel disease-associated variants, common sequence variants, and results from newborn screening. Hum Mutat.

[5] Peruzzo P, Pavan E, Dardis A (2019). Molecular genetics of Pompe disease: a comprehensive overview. Ann Transl Med.

[6] Hernández-Arévalo P, Santotoribio JD, Delarosa-Rodríguez R (2021). Genotype-phenotype correlation of 17 cases of Pompe disease in Spanish patients and identification of 4 novel GAA variants. Orphanet J Rare Dis.

[7] Puri RD, Setia N, N V (2021). Late onset Pompe disease in India - beyond the Caucasian phenotype. Neuromuscul Disord.

[8] Toscano A, Rodolico C, Musumeci O (2019). Multisystem late onset Pompe disease (LOPD): an update on clinical aspects. Ann Transl Med.

[9] Tchan M, Henderson R, Kornberg A (2020). Is it Pompe disease? Australian diagnostic considerations. Neuromuscul Disord.

[10] Al Shehri A, Al-Asmi A, Al Salti AM (2022). A multidisciplinary perspective addressing the diagnostic challenges of late-onset Pompe disease in the Arabian Peninsula region developed from an expert group meeting. J Neuromuscul Dis.

[11] (2023). Outcome and Process Assessment, Health Care. https://www.ncbi.nlm.nih.gov/mesh/68010043.

[12] RDCom SO|., Argüelles CF, Alegre DMI (2023). Assessment tools in Pompe disease: a scoping review protocol. https://osf.io/j7n9c/.

[13] Tricco AC, Lillie E, Zarin W (2018). PRISMA Extension for Scoping Reviews (PRISMA-ScR): checklist and explanation. Ann Intern Med.

[14] (2023). Diagnostic Screening Programs. https://www.ncbi.nlm.nih.gov/mesh/2027878..

[15] (2023). Diagnosis. https://www.ncbi.nlm.nih.gov/mesh/68003933..

[16] (2023). Follow-Up Studies. https://www.ncbi.nlm.nih.gov/mesh/68005500..

[17] Musumeci O, la Marca G, Spada M (2016). LOPED study: looking for an early diagnosis in a late-onset Pompe disease high-risk population. J Neurol Neurosurg Psychiatry.

[18] Confalonieri M, Vitacca M, Scala R (2019). Is early detection of late-onset Pompe disease a pneumologist's affair? A lesson from an Italian screening study. Orphanet J Rare Dis.

[19] (2022). EndNote | The best reference management tool. https://endnote.com/..

[20] (2023). Online, collaborative spreadsheets. https://workspace.google.com/intl/eng/products/sheets/.

[21] (2023). Forms. https://workspace.google.com/intl/eng/lp/forms/..

[22] Peters MD, Godfrey CM, Khalil H, McInerney P, Parker D, Soares CB (2015). Guidance for conducting systematic scoping reviews. Int J Evid Based Healthc.

[23] Tricco AC, Lillie E, Zarin W (2016). A scoping review on the conduct and reporting of scoping reviews. BMC Med Res Methodol.

[24] Kronn DF, Day-Salvatore D, Hwu WL (2017). Management of confirmed newborn-screened patients with Pompe disease across the disease spectrum. Pediatrics.

[25] Bodamer OA, Scott CR, Giugliani R (2017). Newborn screening for Pompe disease. Pediatrics.

[26] Davison JE (2020). Advances in diagnosis and management of Pompe disease. J Mother Child.

[27] Sawada T, Kido J, Nakamura K (2020). Newborn screening for Pompe disease. Int J Neonatal Screen.

[28] Stevens D, Milani-Nejad S, Mozaffar T (2022). Pompe disease: a clinical, diagnostic, and therapeutic overview. Curr Treat Options Neurol.

[29] Burton BK, Kronn DF, Hwu WL, Kishnani PS (2017). The initial evaluation of patients after positive newborn screening: recommended algorithms leading to a confirmed diagnosis of Pompe disease. Pediatrics.

[30] Tortorelli S, Eckerman JS, Orsini JJ (2018). Moonlighting newborn screening markers: the incidental discovery of a second-tier test for Pompe disease. Genet Med.

[31] Hwu WL, Chien YH, Wang R (2021). Newborn screening for Pompe disease. https://www.mdpi.com/books/reprint/4026-newborn-screening-for-pompe-disease.

[32] Al-Hassnan Z, Hashmi NA, Makhseed N, Omran TB, Al Jasmi F, Teneiji AA (2022). Expert Group Consensus on early diagnosis and management of infantile-onset Pompe disease in the Gulf Region. Orphanet J Rare Dis.

[33] Tsai AC, Hung YW, Harding C, Koeller DM, Wang J, Wong LC (2017). Next generation deep sequencing corrects diagnostic pitfalls of traditional molecular approach in a patient with prenatal onset of Pompe disease. Am J Med Genet A.

[34] Ullah A, Zubaida B, Cheema HA, Naeem M (2020). Identification of two novel variants in GAA underlying infantile-onset Pompe disease in two Pakistani families. J Pediatr Endocrinol Metab.

[35] Kishnani PS, Gibson JB, Gambello MJ (2019). Clinical characteristics and genotypes in the ADVANCE baseline data set, a comprehensive cohort of US children and adolescents with Pompe disease. Genet Med.

[36] Bergsma AJ, In 't Groen SL, van den Dorpel JJ (2019). A genetic modifier of symptom onset in Pompe disease. EBioMedicine.

[37] Fernández-Simón E, Carrasco-Rozas A, Gallardo E (2019). PDGF-BB serum levels are decreased in adult onset Pompe patients. Sci Rep.

[38] Liu HX, Pu CQ, Shi Q, Zhang YT, Ban R (2018). Identification of seven novel mutations in the acid alpha-glucosidase gene in five Chinese patients with late-onset Pompe disease. Chin Med J (Engl).

[39] Johnson K, Töpf A, Bertoli M (2017). Identification of GAA variants through whole exome sequencing targeted to a cohort of 606 patients with unexplained limb-girdle muscle weakness. Orphanet J Rare Dis.

[40] Reuser AJ, van der Ploeg AT, Chien YH (2019). GAA variants and phenotypes among 1,079 patients with Pompe disease: data from the Pompe Registry. Hum Mutat.

[41] Blair HA (2023). Cipaglucosidase alfa: first approval. Drugs.

[42] Claeys KG, D'Hondt A, Fache L, Peers K, Depuydt CE (2022). Six-minute walk distance is a useful outcome measure to detect motor decline in treated late-onset Pompe disease patients. Cells.

[43] Figueroa-Bonaparte S, Llauger J, Segovia S (2018). Quantitative muscle MRI to follow up late onset Pompe patients: a prospective study. Sci Rep.

[44] Lai CJ, Hsu TR, Yang CF, Chen SJ, Chuang YC, Niu DM (2016). Cognitive development in infantile-onset Pompe disease under very early enzyme replacement therapy. J Child Neurol.

[45] Yang CF, Yang CC, Liao HC (2016). Very early treatment for infantile-onset Pompe disease contributes to better outcomes. J Pediatr.

[46] Schoser B, Stewart A, Kanters S (2017). Survival and long-term outcomes in late-onset Pompe disease following alglucosidase alfa treatment: a systematic review and meta-analysis. J Neurol.

[47] Navarrete-Martínez JI, Limón-Rojas AE, Gaytán-García MJ (2017). Newborn screening for six lysosomal storage disorders in a cohort of Mexican patients: three-year findings from a screening program in a closed Mexican health system. Mol Genet Metab.

[48] Giugliani R, Castillo Taucher S, Hafez S (2022). Opportunities and challenges for newborn screening and early diagnosis of rare diseases in Latin America. Front Genet.

[49] Kubaski F, Sousa I, Amorim T (2023). Pilot study of newborn screening for six lysosomal diseases in Brazil. Mol Genet Metab.

[50] Kubaski F, Sousa I, Amorim T (2020). Neonatal screening for MPS disorders in Latin America: a survey of pilot initiatives. Int J Neonatal Screen.

[51] Borrajo GJ (2021). Newborn screening in Latin America: a brief overview of the state of the art. Am J Med Genet C Semin Med Genet.

[52] Cabello JF, Novoa F, Huff HV, Colombo M (2021). Expanded newborn screening and genomic sequencing in Latin America and the resulting social justice and ethical considerations. Int J Neonatal Screen.

[53] Wasserstein MP, Orsini JJ, Goldenberg A, Caggana M, Levy PA, Breilyn M, Gelb MH (2021). The future of newborn screening for lysosomal disorders. Neurosci Lett.

[54] Keutzer JM (2020). Establishing Pompe disease newborn screening: the role of industry. Int J Neonatal Screen.

[55] Straub V, Murphy A, Udd B (2018). 229th ENMC international workshop: limb girdle muscular dystrophies - nomenclature and reformed classification Naarden, the Netherlands, 17-19 March 2017. Neuromuscul Disord.

[56] Niño MY, Wijgerde M, de Faria DO (2021). Enzymatic diagnosis of Pompe disease: lessons from 28 years of experience. Eur J Hum Genet.

[57] Goldstein JL, Young SP, Changela M (2009). Screening for Pompe disease using a rapid dried blood spot method: experience of a clinical diagnostic laboratory. Muscle Nerve.

[58] Kishnani PS, Amartino HM, Lindberg C, Miller TM, Wilson A, Keutzer J (2014). Methods of diagnosis of patients with Pompe disease: data from the Pompe Registry. Mol Genet Metab.

[59] Wencel M, Shaibani A, Goyal NA (2021). Investigating late-onset Pompe prevalence in neuromuscular medicine academic practices: the IPaNeMA study. Neurol Genet.

[60] Puzzo F, Colella P, Biferi MG (2017). Rescue of Pompe disease in mice by AAV-mediated liver delivery of secretable acid α-glucosidase. Sci Transl Med.

[61] Kishnani PS, Koeberl DD (2019). Liver depot gene therapy for Pompe disease. Ann Transl Med.

[62] Ronzitti G, Collaud F, Laforet P, Mingozzi F (2019). Progress and challenges of gene therapy for Pompe disease. Ann Transl Med.

[63] Lim JA, Li L, Raben N (2014). Pompe disease: from pathophysiology to therapy and back again. Front Aging Neurosci.

[64] Bekircan-Kurt CE, Güneş HN, Yildiz FG, Saka E, Tan E, Erdem-Özdamar S (2017). New mutations and genotype-phenotype correlation in late-onset Pompe patients. Acta Neurol Belg.

[65] Guevara-Campos J, González-Guevara L, Cauli O (2019). Skeletal alterations, developmental delay and new mutations in juvenile-onset Pompe disease. Neuromuscul Disord.

[66] Niño MY, In 't Groen SL, Bergsma AJ (2019). Extension of the Pompe mutation database by linking disease-associated variants to clinical severity. Hum Mutat.

[67] Málaga DR, Brusius-Facchin AC, Siebert M (2019). Sensitivity, advantages, limitations, and clinical utility of targeted next-generation sequencing panels for the diagnosis of selected lysosomal storage disorders. Genet Mol Biol.

[68] Savarese M, Torella A, Musumeci O (2018). Targeted gene panel screening is an effective tool to identify undiagnosed late onset Pompe disease. Neuromuscul Disord.

[69] Sniderman King L, Pan Y, Nallamilli BR (2023). Pompe disease ascertained through The Lantern Project, 2018-2021: next-generation sequencing and enzymatic testing to overcome obstacles to diagnosis. Mol Genet Metab.

[70] Ebbink BJ, Poelman E, Aarsen FK (2018). Classic infantile Pompe patients approaching adulthood: a cohort study on consequences for the brain. Dev Med Child Neurol.

[71] Kishnani PS, Goldenberg PC, DeArmey SL (2010). Cross-reactive immunologic material status affects treatment outcomes in Pompe disease infants. Mol Genet Metab.

[72] Bali DS, Goldstein JL, Banugaria S, Dai J, Mackey J, Rehder C, Kishnani PS (2012). Predicting cross-reactive immunological material (CRIM) status in Pompe disease using GAA mutations: lessons learned from 10 years of clinical laboratory testing experience. Am J Med Genet C Semin Med Genet.

[73] Wang Z, Okamoto P, Keutzer J (2014). A new assay for fast, reliable CRIM status determination in infantile-onset Pompe disease. Mol Genet Metab.

[74] Kishnani PS, Amartino HM, Lindberg C, Miller TM, Wilson A, Keutzer J (2013). Timing of diagnosis of patients with Pompe disease: data from the Pompe registry. Am J Med Genet A.

[75] Güngör D, Reuser AJ (2013). How to describe the clinical spectrum in Pompe disease?. Am J Med Genet A.

[76] Kishnani PS, Steiner RD, Bali D (2006). Pompe disease diagnosis and management guideline. Genet Med.

[77] Hubig L, Sussex A-K, MacCulloch A (2023). Quality of life with late-onset Pompe disease: qualitative interviews and general public utility estimation in the United Kingdom. J Health Econ Outcomes Res.

[78] Chen S, Wang J, Zhu J, Chung RY, Dong D (2021). Quality of life and its contributors among adults with late-onset Pompe disease in China. Orphanet J Rare Dis.

[79] Hughes D, Odedra K, Bashorum L (2022). Living with Pompe disease in the UK: characterizing the patient journey and burden on physical, emotional and social quality of life. Mol Genet Metab.

[80] Schoser B, Bilder DA, Dimmock D, Gupta D, James ES, Prasad S (2017). The humanistic burden of Pompe disease: are there still unmet needs? A systematic review. BMC Neurol.

[81] Kanters TA, van der Ploeg AT, Brouwer WB, Hakkaart L (2013). The impact of informal care for patients with Pompe disease: an application of the CarerQol instrument. Mol Genet Metab.

[82] Li D, Lin Y, Huang Y (20182024). Early prenatal diagnosis of lysosomal storage disorders by enzymatic and molecular analysis. Prenat Diagn [Internet.

[83] Rairikar MV, Case LE, Bailey LA (20172024). Insight into the phenotype of infants with Pompe disease identified by newborn screening with the common c.-32-13T>G “late-onset” GAA variant. Mol Genet Metab [Internet.

[84] Chen X, Liu T, Huang M (20172024). Clinical and molecular characterization of infantile-onset Pompe disease in mainland Chinese patients: Identification of two common mutations. Genet Test Mol Biomarkers [Internet.

[85] Moravej H, Amirhakimi A, Showraki A, Amoozgar H, Hadipour Z, Nikfar G (20182024). A new mutation causing severe infantile-onset Pompe disease responsive to enzyme replacement therapy. Iran J Med Sci [Internet.

[86] Semplicini C, Letard P, De Antonio M (20182024). Late-onset Pompe disease in France: molecular features and epidemiology from a nationwide study. J Inherit Metab Dis [Internet.

[87] Herbert M, Cope H, Li JS, Kishnani PS (20182024). Severe cardiac involvement is rare in patients with late-onset Pompe disease and the common c.-32-13T>G variant: Implications for newborn screening. J Pediatr [Internet.

[88] Torrealba-Acosta G, Rodríguez-Roblero MC, Bogantes-Ledezma S, Carazo-Céspedes K, Desnuelle C (20172024). First clinical and genetic description of a family diagnosed with late-onset Pompe disease from Costa Rica. Neuromuscul Disord [Internet.

[89] Ebrahimi M, Behnam M, Behranvand-Jazi N (2017). Identification a novel mononucleotide deletion mutation in GAA in pompe disease patients. J Res Med Sci [Internet.

